# Supplementary material for: The role of cerebral blood flow volume in cortical inhibition during postural changes
Source: PeerJ. 2025 Oct 27;13:e20233. doi: 10.7717/peerj.20233 (PMC12574591; doi:10.7717/peerj.20233)
Supplement: Supplemental Information 1 — nnd –non-Gaussian distribution, ns –insignificant results. The table demonstrates achieved effect size for each parameter that had significant changes in both tests. Values of achieved effect size (fa) that are equal or exceed the value of correspondent required effect size (fr) calculated for every sample are highlighted by green color. [file peerj-13-20233-s001.docx]

**Supplemental Table 1:**

**Achieved effect size (*f_a_*) for total sample in both tests.**

nnd – non-Gaussian distribution, ns – insignificant results. The table demonstrates achieved effect size for each parameter that had significant changes in both tests. Values of achieved effect size (*f_a_*) that are equal or exceed the value of correspondent required effect size (*f_r_*) calculated for every sample are highlighted by bold text.

| Parameters | Test 1 | Test 2 |
| --- | --- | --- |
| Pα in F3 | **0.29** | 0.19 |
| Pα in F4 | **0.28** | 0.16 |
| Pα in F7 | **0.23** | ns |
| Pα in F8 | nnd | ns |
| Pα in C3 | nnd | 0.18 |
| Pα in C4 | **0.29** | **0.23** |
| Pα in T3 | **0.23** | 0.14 |
| Pα in T4 | **0.27** | ns |
| Pα in T5 | nnd | 0.14 |
| Pα in T6 | nnd | nnd |
| Pα in P3 | nnd | nnd |
| Pα in P4 | nnd | ns |
| Pα in Fz | **0.28** | 0.17 |
| Pα in Cz | **0.34** | **0.21** |
| Pα in Pz | **0.34** | 0.17 |
| RWA in LFM | 0.12 | 0.2 |
| RWA in RFM | 0.18 | **0.26** |
| RWA in LOM | 0.07 | nnd |
| RWA in ROM | 0.07 | nnd |
| VO in LFM | **0.38** | **0.4** |
| VO in RFM | **0.22** | 0.12 |
| VO in LOM | **0.22** | nnd |
| VO in ROM | nnd | 0.122 |
| CT in LFM | **0.6** | **0.59** |
| CT in RFM | **0.51** | **0.43** |
| CT in LOM | **0.43** | **0.48** |
| CT in ROM | **0.31** | ns |
