# Supplementary material for: The role of cerebral blood flow volume in cortical inhibition during postural changes
Source: PeerJ. 2025 Oct 27;13:e20233. doi: 10.7717/peerj.20233 (PMC12574591; doi:10.7717/peerj.20233)
Supplement: Supplemental Information 4 — All samples were normally distributed. M and SD are mean value and standard deviation. Asterisk (*) points out the values in horizontal supine position that significantly differed from sitting upright (p < 0.05). The underlined values indicate statistical difference between them in two horizontal supine positions or within the same horizontal supine position (p < 0,05). [file peerj-13-20233-s004.docx]

**Supplemental Table 4:**

**SAP, DAP and heart rate changes during different body position in Test 1.**

All samples were normally distributed. M and SD are mean value and standard deviation. Asterisk (*) points out the values in horizontal supine position that significantly differed from sitting upright (p < 0.05). The underlined values indicate statistical difference between them in two horizontal supine positions or within the same horizontal supine position (p < 0,05).

| Statistical indexes | First sitting upright | | Horizontal supine (first 2 minutes) | | Horizontal supine (last 2 minutes) | | Second sitting upright | |
| --- | --- | --- | --- | --- | --- | --- | --- | --- |
|  | Male | Female | Male | Female | Male | Female | Male | Female |
|  | **Postural changes in SAP** | | | | | | | |
| M | 119.8 | 112.3 | 119.7 | 109.7 | 115.9 | 105.4* | 119.7 | 110.5 |
| SD | 9.35 | 6.5 | 8.1 | 8.79 | 7.57 | 6.79 | 9.95 | 7.78 |
|  | **Postural changes in DAP** | | | | | | | |
| M | 73.05 | 75.14 | 64.24* | 66.91* | 63.33* | 66.41* | 73.57 | 76.23 |
| SD | 6.11 | 7.46 | 7.52 | 6.02 | 6.07 | 6.6 | 6.32 | 6.73 |
|  | **Postural changes in heart rate** | | | | | | | |
| M | 77.00 | 80.09 | 65.33* | 71.55* | 68.29* | 70.50* | 76.81 | 79.86 |
| SD | 9.767 | 16.41 | 10.75 | 16.54 | 13.44 | 14.50 | 11.45 | 15.19 |
