# Supplementary material for: The role of cerebral blood flow volume in cortical inhibition during postural changes
Source: PeerJ. 2025 Oct 27;13:e20233. doi: 10.7717/peerj.20233 (PMC12574591; doi:10.7717/peerj.20233)
Supplement: Supplemental Information 5 — All samples were normally distributed. M and SD are mean value and standard deviation. Asterisk (*) points out the values in horizontal supine position that significantly differed from sitting upright (p < 0.05). The underlined values indicate statistical difference between them in two horizontal supine positions or within the same horizontal supine position (p < 0.05). [file peerj-13-20233-s005.docx]

**Supplementary Table 5:**

**SAP, DAP and heart rate changes during different body position in Test 2.**

All samples were normally distributed. M and SD are mean value and standard deviation. Asterisk (*) points out the values in horizontal supine position that significantly differed from sitting upright (*p* < 0.05). The underlined values indicate statistical difference between them in two horizontal supine positions or within the same horizontal supine position (*p* < 0.05).

| Statistical indexes | First sitting upright | | First horizontal supine | | Second horizontal supine | | Second sitting upright | |
| --- | --- | --- | --- | --- | --- | --- | --- | --- |
|  | Male | Female | Male | Female | Male | Female | Male | Female |
|  | **Postural changes in SAP** | | | | | | | |
| M | 115.8 | 110.4 | 119.2 | 109.2 | 115.8* | 107.4 | 119.6 | 109.8 |
| SD | 10.29 | 8.1 | 9.25 | 8.55 | 7.74 | 8.48 | 9.97 | 8.77 |
|  | **Postural changes in DAP** | | | | | | | |
| M | 72.33 | 74.23 | 62.89* | 68.00* | 64.06* | 67.41* | 75.39 | 76.41 |
| SD | 7.71 | 6.7 | 8.13 | 5.45 | 8.91 | 6.8 | 7.81 | 6.67 |
|  | **Postural changes in heart rate** | | | | | | | |
| M | 76.44 | 77.91 | 64.22* | 70.91* | 66.56* | 69.73* | 77.22 | 77.59 |
| SD | 10.23 | 15.72 | 9.849 | 16.20 | 9.275 | 13.06 | 10.21 | 14.74 |
