# Supplementary material for: The role of cerebral blood flow volume in cortical inhibition during postural changes
Source: PeerJ. 2025 Oct 27;13:e20233. doi: 10.7717/peerj.20233 (PMC12574591; doi:10.7717/peerj.20233)
Supplement: Supplemental Information 6 — LFM –left FM, RFM –right FM, LOM –left OM, ROM –right OM. r –Spearman’s correlation coefficient, p –statistical significance. Statistically significant results are highlighted by green color. The value of n equals to the number of pairs of averaged RWA and Pα between participants in each analyzed sample of EEG and REG (oSA1, oSA2, oHA1, etc.). [file peerj-13-20233-s006.docx]

**Suplemental Table 6:**

**Correlation results between RWA and Pα in Test 2 (*n* = 8).**

LFM – left FM, RFM – right FM, LOM – left OM, ROM – right OM. *r* – Spearman’s correlation coefficient, *p* – statistical significance. Statistically significant results are highlighted by bold text. The value of *n* equals to the number of pairs of averaged RWA and Pα between participants in each analyzed sample of EEG and REG (oSA1, oSA2, oHA1, etc.).

| Рα  RWA | | Males | | | | | Females | | | | |
| --- | --- | --- | --- | --- | --- | --- | --- | --- | --- | --- | --- |
|  |  | F3 | F7 | C3 | P3 | T5 | F3 | F7 | C3 | P3 | T5 |
| LFM | *r* | -0,6228 | -0,6228 | -0,4551 | -0,5509 | -0,527 | -0,2857 | -0,1190 | -0,3810 | -0,3095 | -0,3333 |
|  | *p* | 0,105 | 0,105 | 0,2599 | 0,1634 | 0,1847 | 0,5008 | 0,7930 | 0,3599 | 0,4618 | 0,4279 |
| LOM | *r* | 0 | 0 | 0,2755 | 0,2994 | 0 | -0,3114 | -0,1677 | **-0,7545** | -0,2874 | -0,5270 |
|  | *p* | >0,999 | >0,999 | 0,5076 | 0,4714 | >0,999 | 0,4475 | 0,6932 | **0,0380** | 0,4844 | 0,1846 |
|  | | F4 | F8 | C4 | P4 | T6 | F4 | F8 | C4 | P4 | T6 |
| RFM | *r* | -0,7143 | -0,619 | -0,7143 | -0,6667 | -0,619 | -0,3353 | -0,1078 | -0,6587 | -0,5389 | -0,4311 |
|  | *p* | 0,0576 | 0,115 | 0,0576 | 0,0831 | 0,115 | 0,4104 | 0,8063 | 0,0850 | 0,1765 | 0,2854 |
| ROM | *r* | **0,8571** | 0,5714 | **0,8571** | **0,8095** | 0,5238 | -0,3593 | -0,5988 | -0,5389 | -0,6587 | -0,2275 |
|  | *p* | **0,0107** | 0,1511 | **0,0107** | **0,0218** | 0,1966 | 0,3786 | 0,1248 | 0,1765 | 0,0850 | 0,5859 |
