# Supplementary material for: The role of cerebral blood flow volume in cortical inhibition during postural changes
Source: PeerJ. 2025 Oct 27;13:e20233. doi: 10.7717/peerj.20233 (PMC12574591; doi:10.7717/peerj.20233)
Supplement: Supplemental Information 7 — LFM –left FM, RFM –right FM, LOM –left OM, ROM –right OM. r –Spearman’s correlation coefficient, p –statistical significance. Statistically significant results are highlighted by green color. The value of n equals to the number of pairs of averaged RWA and Pα between participants in each analyzed sample of EEG and REG (oSA1, oSA2, oHA1, etc.). [file peerj-13-20233-s007.docx]

**Suplemental Table 7:**

**Correlation results between RWA and Pα among all participants in both tests (*n* = 8).**

LFM – left FM, RFM – right FM, LOM – left OM, ROM – right OM. *r* – Spearman’s correlation coefficient, *p* – statistical significance. Statistically significant results are highlighted by bold text. The value of *n* equals to the number of pairs of averaged RWA and Pα between participants in each analyzed sample of EEG and REG (oSA1, oSA2, oHA1, etc.).

| Рα  RWA | | Test 1 | | | | | Test 2 | | | | |
| --- | --- | --- | --- | --- | --- | --- | --- | --- | --- | --- | --- |
|  |  | F3 | F7 | C3 | P3 | T5 | F3 | F7 | C3 | P3 | T5 |
| LFM | *r* | **-0,7785** | **-0,7785** | **-0,8144** | **-0,7904** | **-0,7904** | -0,619 | -0,5952 | -0,5238 | -0,3571 | -0,3571 |
|  | *p* | **0,0295** | **0,0295** | **0,0184** | **0,0248** | **0,0248** | 0,115 | 0,1323 | 0,1966 | 0,3894 | 0,3894 |
| LOM | *r* | -0,0714 | -0,0714 | -0,0238 | -0,0476 | -0,0476 | -0,381 | -0,4048 | -0,4524 | -0,3571 | -0,3571 |
|  | *p* | 0,882 | 0,882 | 0,9768 | 0,9349 | 0,9349 | 0,3599 | 0,3268 | 0,2675 | 0,3894 | 0,3894 |
|  | | F4 | F8 | C4 | P4 | T6 | F4 | F8 | C4 | P4 | T6 |
| RFM | *r* | -0,6905 | -0,6905 | -0,6905 | **-0,7857** | **-0,7857** | -0,5389 | -0,0838 | **-0,7306** | -0,515 | -0,5389 |
|  | *p* | 0,0694 | 0,0694 | 0,0694 | **0,0279** | **0,0279** | 0,1769 | 0,8493 | **0,0467** | 0,1967 | 0,1769 |
| ROM | *r* | **-0,8264** | **-0,8264** | **-0,8264** | **-0,7545** | **-0,7545** | 0,2857 | 0,1905 | 0,3571 | 0,1905 | 0,5952 |
|  | *p* | **0,0163** | **0,0163** | **0,0163** | **0,0377** | **0,0377** | 0,5008 | 0,6646 | 0,3894 | 0,6646 | 0,1323 |
