# Supplementary material for: The role of cerebral blood flow volume in cortical inhibition during postural changes
Source: PeerJ. 2025 Oct 27;13:e20233. doi: 10.7717/peerj.20233 (PMC12574591; doi:10.7717/peerj.20233)
Supplement: Supplemental Information 8 — Horizontal dashed line represents isoline, and vertical dashed lines are the parameters explored in this study. Av and A3/4are main components in estimation of venous output (VO). Anacrotic (AT) and catacrotic (CT) time are also shown as arrowed lines. RWA –reowave amplitude, Av –amplitude of the maximum systolic value of REG wave venous component, A3/4 –reowave amplitude in the last quarter of cardiac cycle. [file peerj-13-20233-s008.pdf]

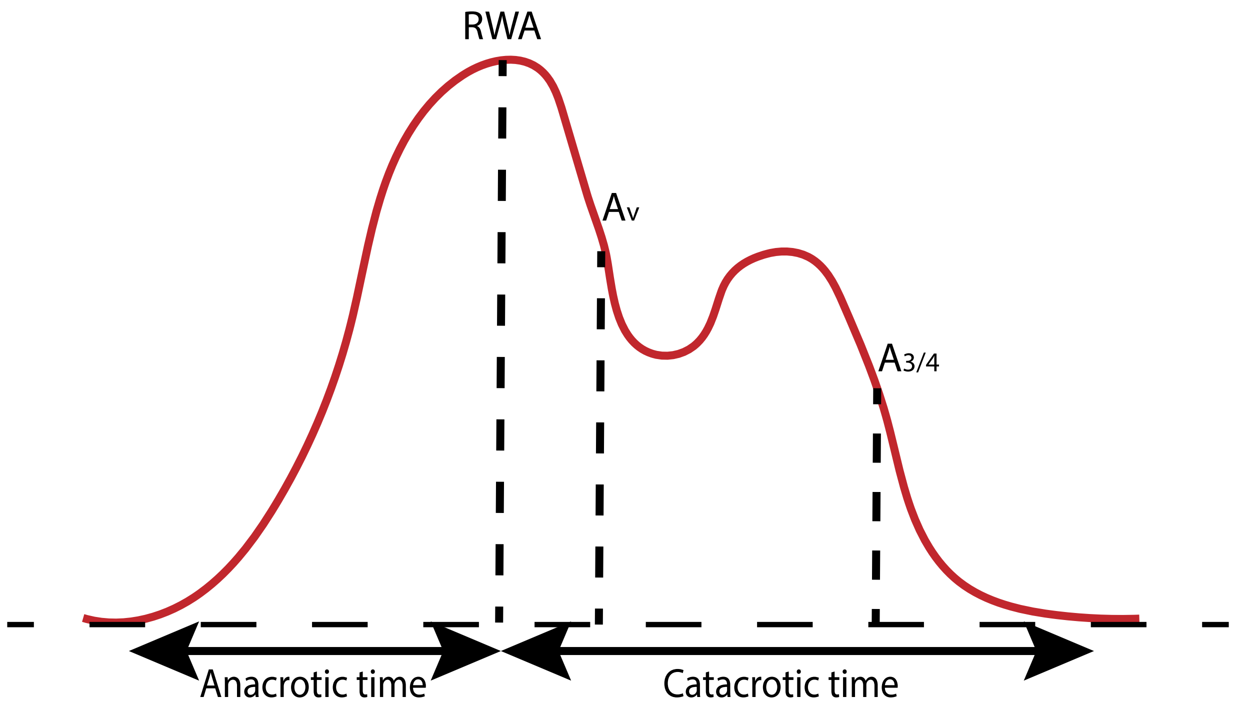

Supplemental Figure 1. The studied parameters of REG wave. Horizontal dashed line represents isoline, and vertical dashed lines are the parameters explored in this study. A_v and $A_{3/4}$ are main components in estimation of venous output (VO). Anacrotic (AT) and catacrotic (CT) time are also shown as arrowed lines. RWA – reowave amplitude, A_v – amplitude of the maximum systolic value of REG wave venous component, $A_{3/4}$ – reowave amplitude in the last quarter of cardiac cycle.
