# Supplementary material for: The role of cerebral blood flow volume in cortical inhibition during postural changes
Source: PeerJ. 2025 Oct 27;13:e20233. doi: 10.7717/peerj.20233 (PMC12574591; doi:10.7717/peerj.20233)
Supplement: Supplemental Information 10 — The graphs show data from 4 REG leads: left and right fronto-mastoid (FM), left and right occcipito-mastoid (OM) for sitting and supine positions. The graphs show confidence intervals with means represented by circle-shaped points, and medians depicted as rhomb-shaped points. Additionally, points and intervals are highlighted by different colors to distinguish between first sitting (SA) and first 2 min of supine (HA) position and second sitting (SB) and last 2 min of supine (HB) position. [file peerj-13-20233-s010.pdf]

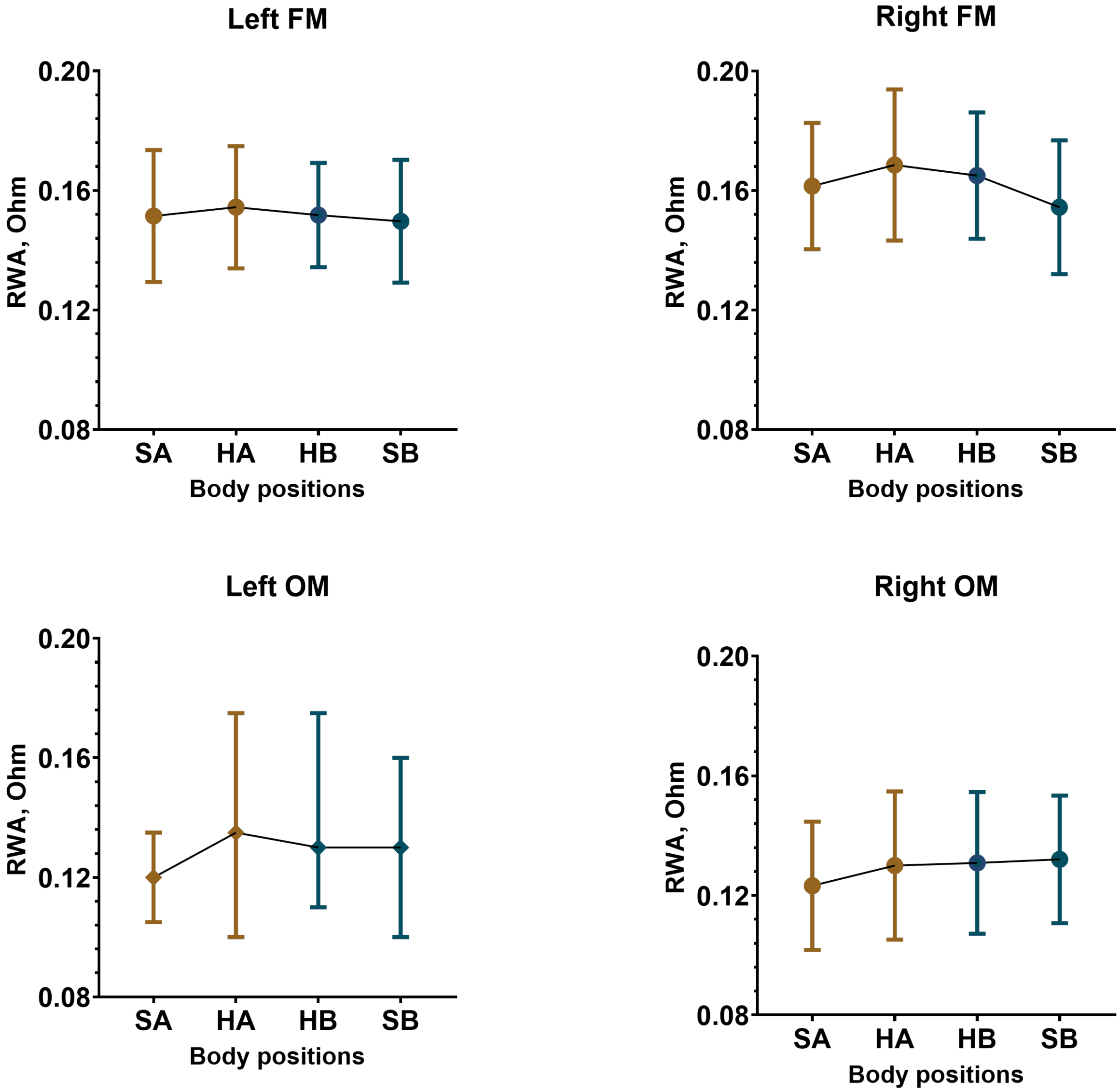

Supplemental Figure 3 (n = 17). Postural changes of RWA among female participants during Test 1. The graphs show data from 4 REG leads: left and right fronto-mastoid (FM), left and right occcipito-mastoid (OM) for sitting and supine positions. The graphs show confidence intervals with means represented by circle-shaped points, and medians depicted as rhomb-shaped points. Additionally, points and intervals are highlighted by different colors to distinguish between first sitting (SA) and first 2 minutes of supine (HA) position and second sitting (SB) and last 2 minutes of supine (HB) position.
