# Supplementary material for: The role of cerebral blood flow volume in cortical inhibition during postural changes
Source: PeerJ. 2025 Oct 27;13:e20233. doi: 10.7717/peerj.20233 (PMC12574591; doi:10.7717/peerj.20233)
Supplement: Supplemental Information 12 — The graphs show data from 4 REG leads: left and right fronto-mastoid (FM), left and right occcipito-mastoid (OM) for sitting and supine positions. The graphs show confidence intervals with means represented by circle-shaped points, and medians depicted as rhomb-shaped points. Additionally, points and intervals are highlighted by different colors to distinguish between first sitting (oSA) and supine (oHA) positions and second sitting (oSB) and supine (oHB) positions. A one-way repeated measures ANOVA summary for statistically significant results: left FM (F (1.348, 20.23) = 5.213, p = 0.0247), right FM (F (1.583, 23.74) = 12.83, p = 0.0004). “*” –p < 0.05, “**” –p < 0.01, “***” –p < 0.001. [file peerj-13-20233-s012.pdf]

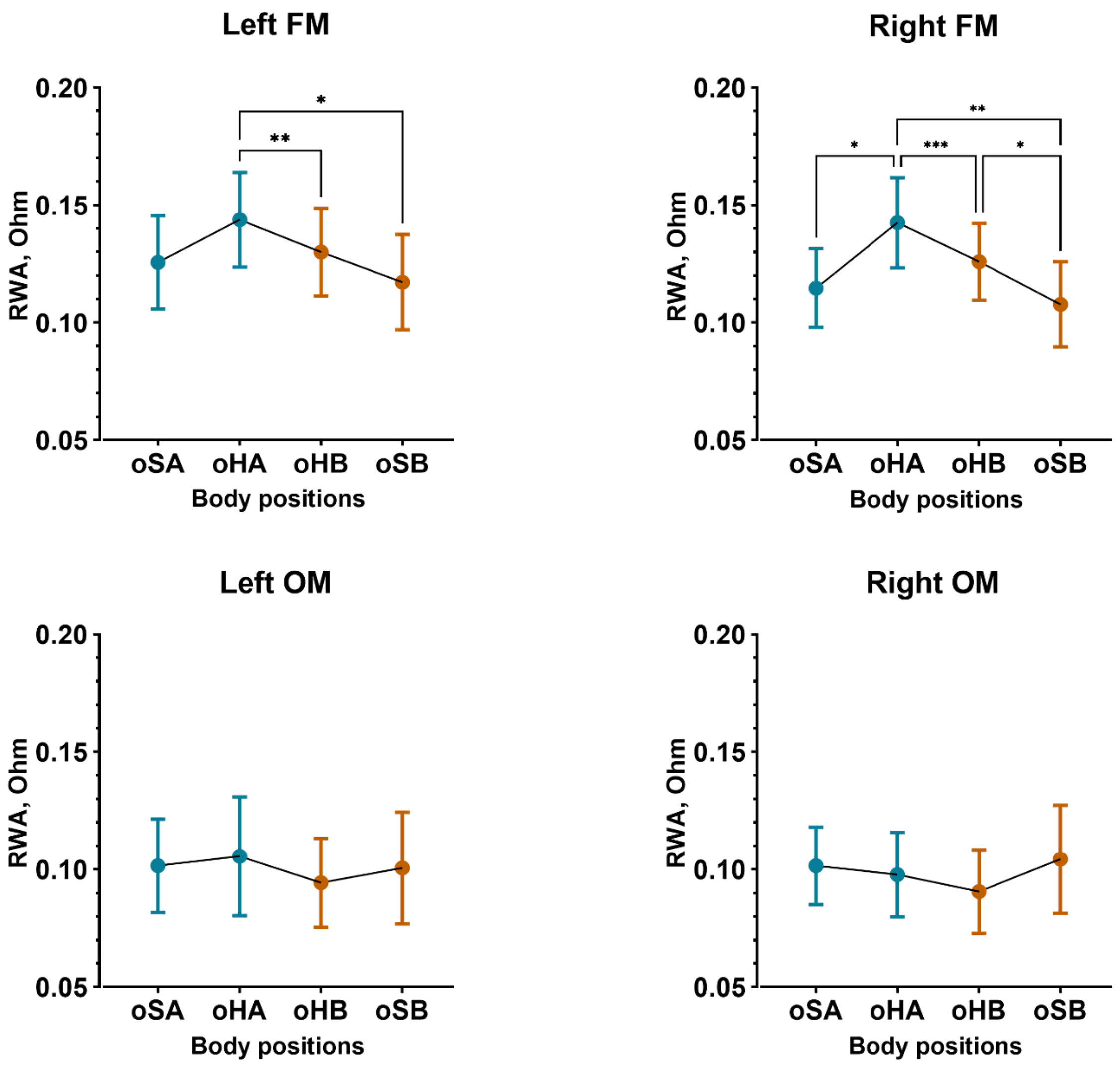

Supplemental Figure 5. Postural changes of RWA among male participants during Test 2 (n = 16). The graphs show data from 4 REG leads: left and right fronto-mastoid (FM), left and right occcipito-mastoid (OM) for sitting and supine positions. The graphs show confidence intervals with means represented by circle-shaped points, and medians depicted as rhomb-shaped points. Additionally, points and intervals are highlighted by different colors to distinguish between first sitting (oSA) and supine (oHA) positions and second sitting (oSB) and supine (oHB) positions. A one-way repeated measures ANOVA summary for statistically significant results: left FM (F (1.348, 20.23) = 5.213, $p = 0.0247$), right FM (F (1.583, 23.74) = 12.83, $p = 0.0004$). "*" – $p < 0.05$, "**" – $p < 0.01$, "***" – $p < 0.001$.
