# Supplementary material for: The role of cerebral blood flow volume in cortical inhibition during postural changes
Source: PeerJ. 2025 Oct 27;13:e20233. doi: 10.7717/peerj.20233 (PMC12574591; doi:10.7717/peerj.20233)
Supplement: Supplemental Information 14 — The graphs show data from 4 REG leads: left and right fronto-mastoid (FM), left and right occcipito-mastoid (OM) for sitting and supine positions. The graphs show confidence intervals with means represented by circle-shaped points, and medians depicted as rhomb-shaped points. Additionally, points and intervals are highlighted by different colors to distinguish between first sitting (oSA) and supine (oHA) positions and second sitting (oSB) and supine (oHB) positions. A one-way repeated measures ANOVA summary for statistically significant results: left FM (F (1.949, 60.42) = 8.408, p = 0.0007), right FM (F (2.101, 60.92) = 15.55, p < 0.0001). “*” –p < 0.05, “***” –p < 0.001, “****” –p < 0.0001. [file peerj-13-20233-s014.pdf]

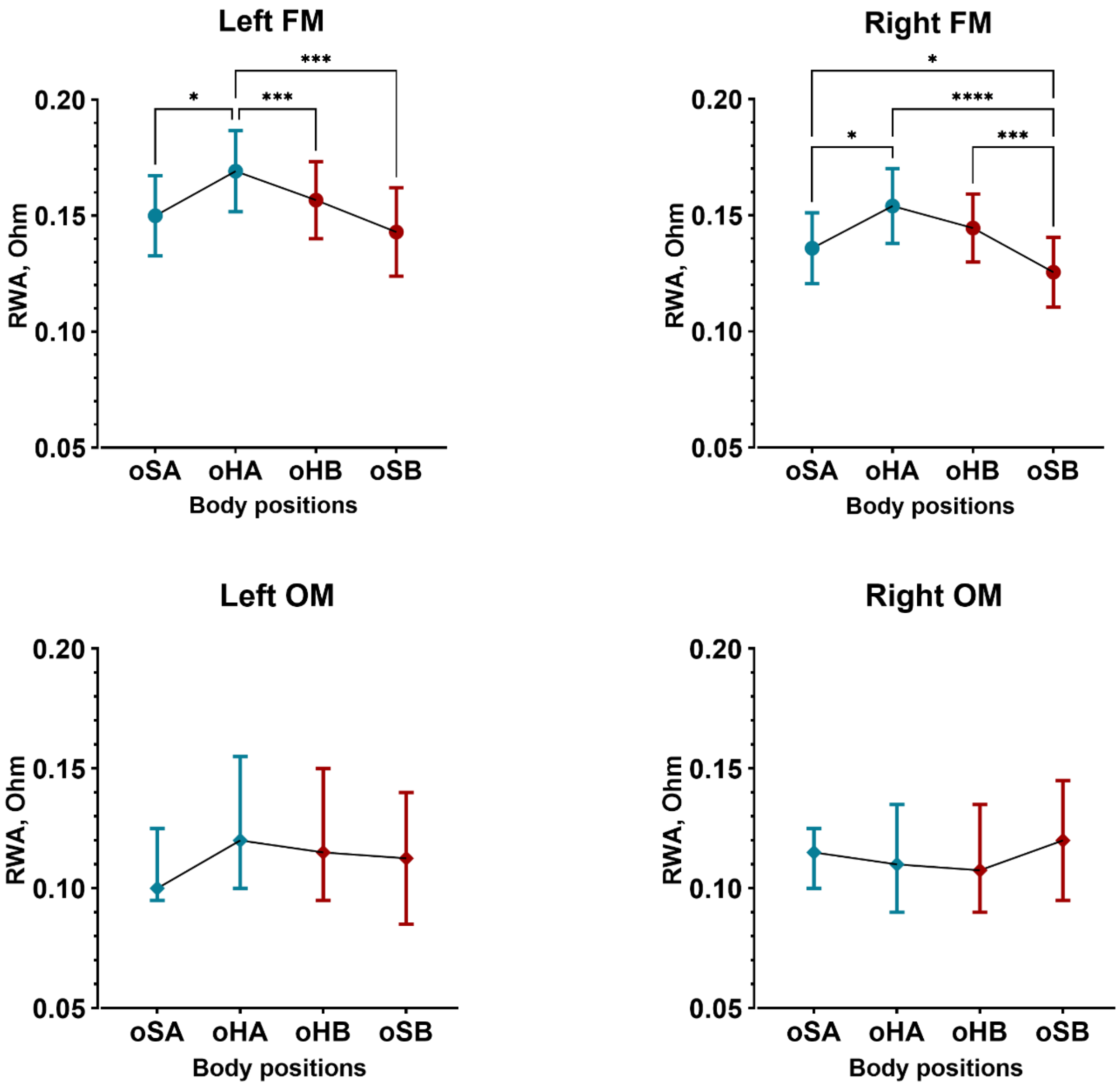

Supplemental Figure 7. Postural changes of RWA among all participants during Test 2 (n = 32). The graphs show data from 4 REG leads: left and right fronto-mastoid (FM), left and right occcipito-mastoid (OM) for sitting and supine positions. The graphs show confidence intervals with means represented by circle-shaped points, and medians depicted as rhomb-shaped points. Additionally, points and intervals are highlighted by different colors to distinguish between first sitting (oSA) and supine (oHA) positions and second sitting (oSB) and supine (oHB) positions. A one-way repeated measures ANOVA summary for statistically significant results: left FM (F (1.949, 60.42) = 8.408, p = 0.0007), right FM (F (2.101, 60.92) = 15.55, p < 0.0001). "*" – p < 0.05, "***" – p < 0.001, "****" – p < 0.0001.
