# Supplementary material for: The role of cerebral blood flow volume in cortical inhibition during postural changes
Source: PeerJ. 2025 Oct 27;13:e20233. doi: 10.7717/peerj.20233 (PMC12574591; doi:10.7717/peerj.20233)
Supplement: Supplemental Information 18 — The graphs show data from 4 REG leads: left and right fronto-mastoid (FM), left and right occcipito-mastoid (OM) for two supine positions (oHA and oHB). Black boxplots include values of male participants (m), and red boxplots contain values of female participants (f). Pairs of boxplots were analyzed separately using one-way ANOVA, i.e., oHA (m) was compared only to oHA (f), and oHB (m) was compared only to oHB (f). A one-way ANOVA summary for statistically significant results: left FM (F (3, 60) = 9.697, p < 0.0001), right FM (F (3, 60) = 5.838, p = 0.0014), left OM (F (3, 60) = 9.511, p < 0.0001), right OM (F (3, 60) = 5.145, p = 0.0031). “*” –p < 0.05, “**” –p < 0.01, “***” –p < 0.001. [file peerj-13-20233-s018.pdf]

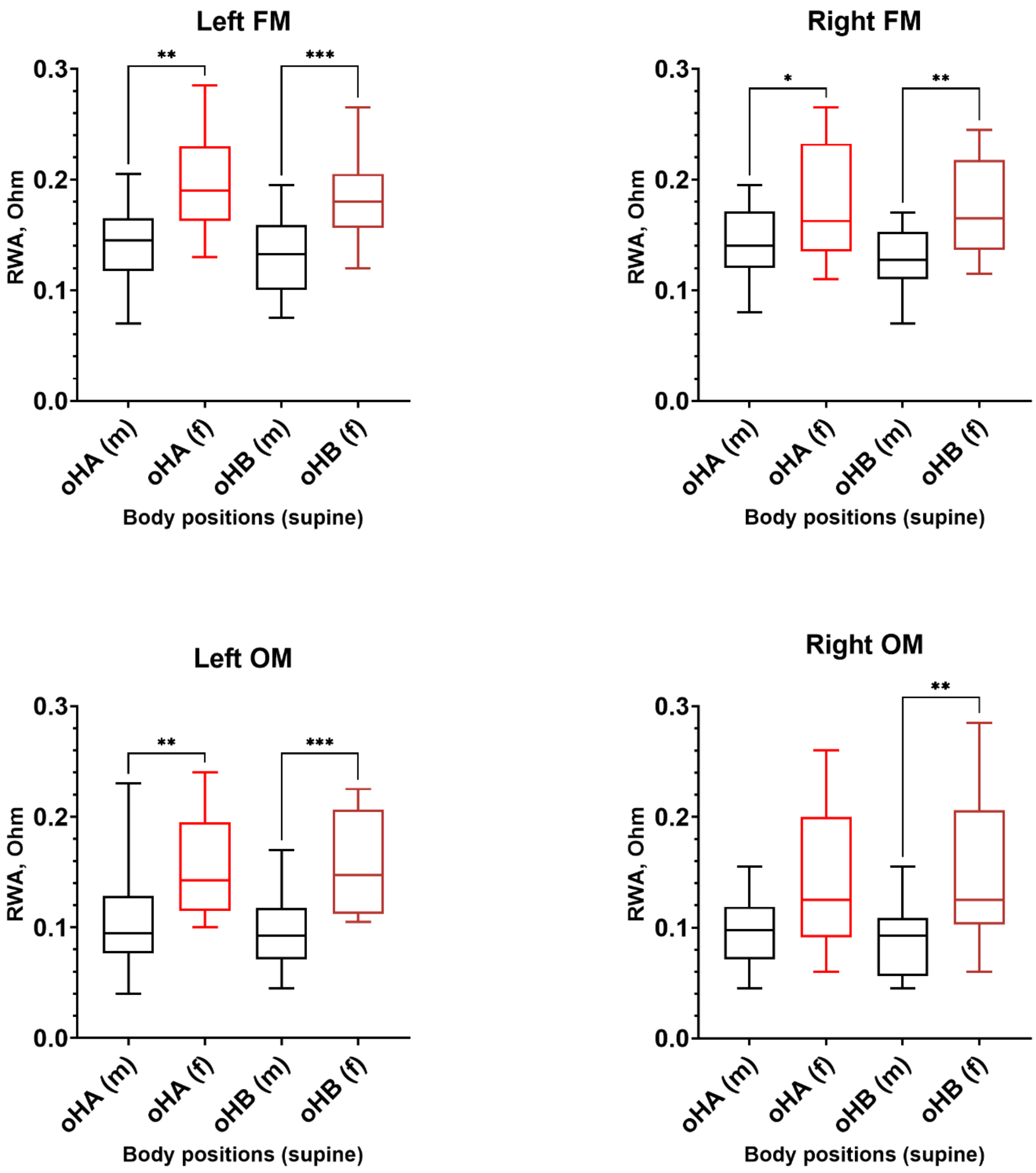

Supplemental Figure 11. Sex differences in RWA during supine positions in Test 2 (*n* = 32). The graphs show data from 4 REG leads: left and right fronto-mastoid (FM), left and right occcipito-mastoid (OM) for two supine positions (oHA and oHB). Black boxplots include values of male participants (m), and red boxplots contain values of female participants (f). Pairs of boxplots were analyzed separately using one-way ANOVA, i.e., oHA (m) was compared only to oHA (f), and oHB (m) was compared only to oHB (f). A one-way ANOVA summary for statistically significant results: left FM (F (3, 60) = 9.697, $p < 0.0001$), right FM (F (3, 60) = 5.838, $p = 0.0014$), left OM (F (3, 60) = 9.511, $p < 0.0001$), right OM (F (3, 60) = 5.145, $p = 0.0031$). "*" – $p < 0.05$, "**" – $p < 0.01$, "***" – $p < 0.001$.
