# Supplementary material for: The role of cerebral blood flow volume in cortical inhibition during postural changes
Source: PeerJ. 2025 Oct 27;13:e20233. doi: 10.7717/peerj.20233 (PMC12574591; doi:10.7717/peerj.20233)
Supplement: Supplemental Information 20 — The graphs show data from 4 REG leads: left and right fronto-mastoid (FM), left and right occcipito-mastoid (OM) for sitting and supine positions. The graphs show confidence intervals with means represented by circle-shaped points, and medians depicted as rhomb-shaped points. Additionally, points and intervals are highlighted by different colors to distinguish between first sitting (SA) and first 2 min of supine (HA) position and second sitting (SB) and last 2 min of supine (HB) position. A one-way repeated measures ANOVA summary for statistically significant results: left FM (F (1.824, 29.19) = 6.621, p = 0.0052). “*” –p < 0.05, “**” –p < 0, 01. [file peerj-13-20233-s020.pdf]

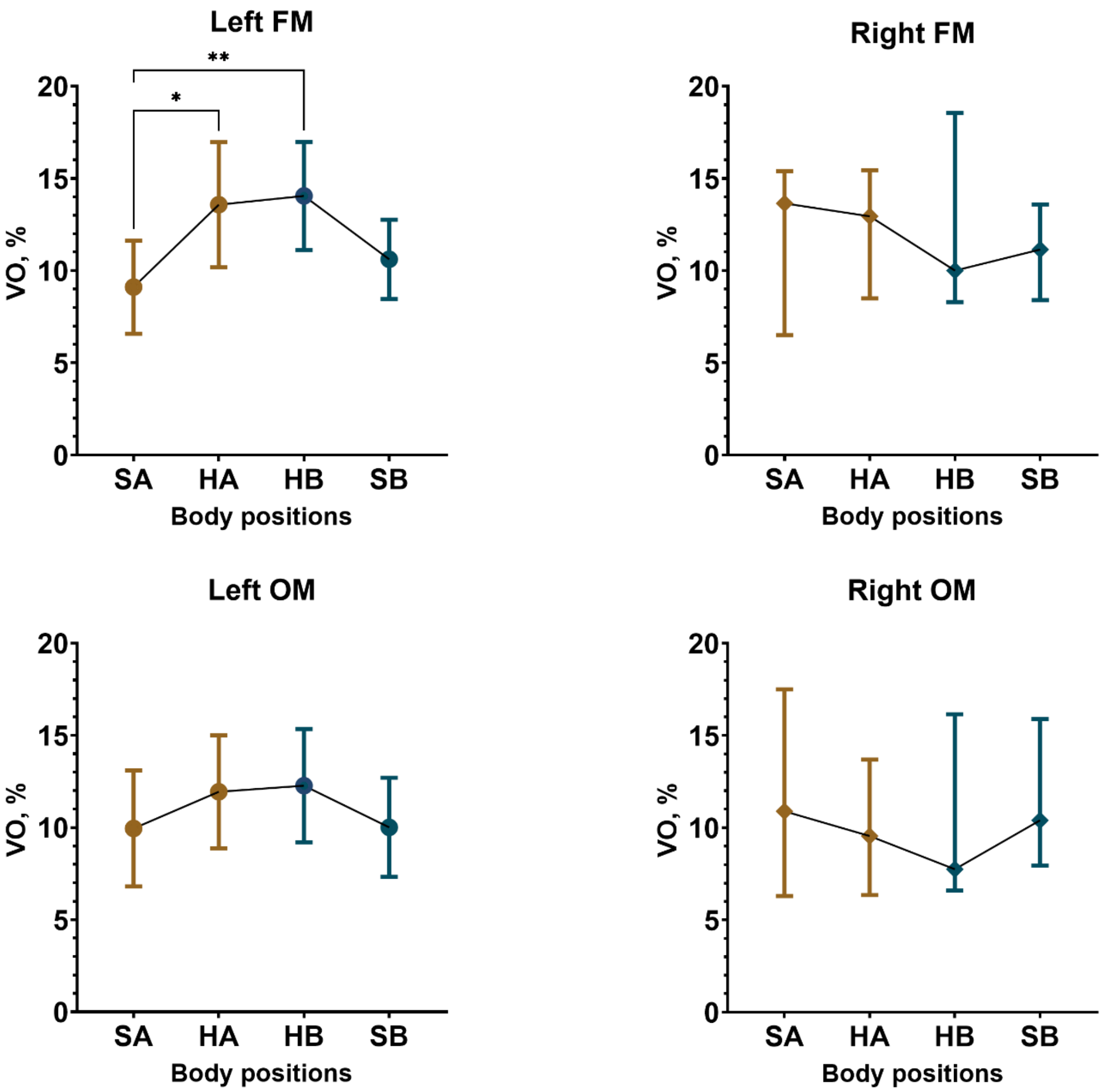

Supplemental Figure 13. Postural changes of VO among female participants during Test 1 (n = 17). The graphs show data from 4 REG leads: left and right fronto-mastoid (FM), left and right occcipito-mastoid (OM) for sitting and supine positions. The graphs show confidence intervals with means represented by circle-shaped points, and medians depicted as rhomb-shaped points. Additionally, points and intervals are highlighted by different colors to distinguish between first sitting (SA) and first 2 minutes of supine (HA) position and second sitting (SB) and last 2 minutes of supine (HB) position. A one-way repeated measures ANOVA summary for statistically significant results: left FM (F (1.824, 29.19) = 6.621, p = 0.0052). "*" – $p < 0.05$, "**" – p < 0,01.
