# Supplementary material for: The role of cerebral blood flow volume in cortical inhibition during postural changes
Source: PeerJ. 2025 Oct 27;13:e20233. doi: 10.7717/peerj.20233 (PMC12574591; doi:10.7717/peerj.20233)
Supplement: Supplemental Information 21 — The graphs show data from 4 REG leads: left and right fronto-mastoid (FM), left and right occcipito-mastoid (OM) for sitting and supine positions. The graphs show confidence intervals with means represented by circle-shaped points, and medians depicted as rhomb-shaped points. Additionally, points and intervals are highlighted by different colors to distinguish between first sitting (SA) and first 2 min of supine (HA) position and second sitting (SB) and last 2 min of supine (HB) position. A one-way repeated measures ANOVA summary for statistically significant results: left FM (F (1.756, 63.23) = 16.34, p < 0.0001). “*” –p < 0.05, “**” –p < 0, 01, “***” –p < 0.001, “****” –p < 0.0001. [file peerj-13-20233-s021.pdf]

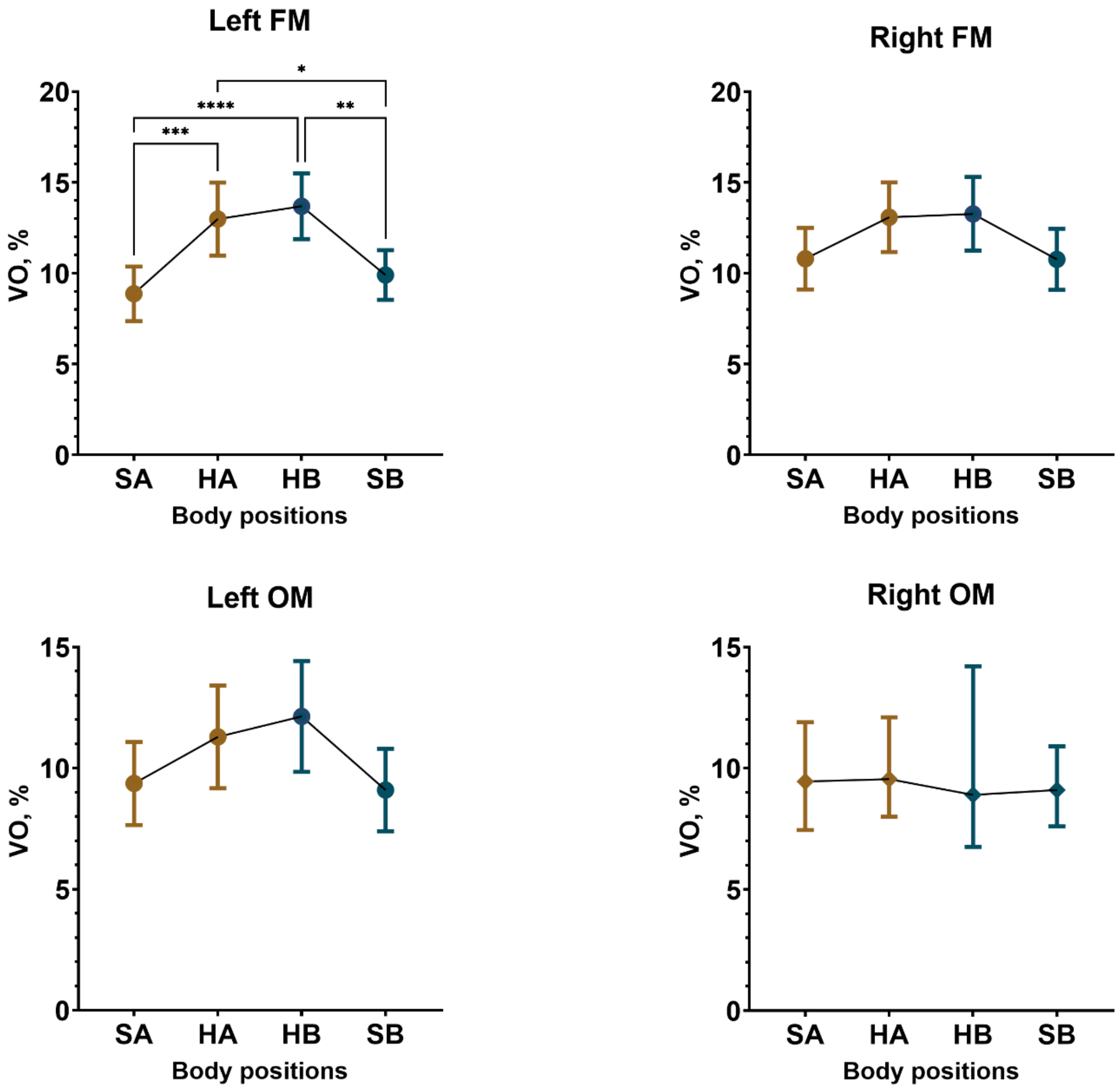


**Supplemental Figure 14. Postural changes of VO among all participants during Test 1 (n = 37).** The graphs show data from 4 REG leads: left and right fronto-mastoid (FM), left and right occcipito-mastoid (OM) for sitting and supine positions. The graphs show confidence intervals with means represented by circle-shaped points, and medians depicted as rhomb-shaped points. Additionally, points and intervals are highlighted by different colors to distinguish between first sitting (SA) and first 2 minutes of supine (HA) position and second sitting (SB) and last 2 minutes of supine (HB) position. A one-way repeated measures ANOVA summary for statistically significant results: left FM ($F$ (1.756, 63.23) = 16.34, $p < 0.0001$). "*" – $p < 0.05$, "**" – p < 0,01, "***" – $p < 0.001$, "****" – $p < 0.0001$.
