# Supplementary material for: The role of cerebral blood flow volume in cortical inhibition during postural changes
Source: PeerJ. 2025 Oct 27;13:e20233. doi: 10.7717/peerj.20233 (PMC12574591; doi:10.7717/peerj.20233)
Supplement: Supplemental Information 24 — The graphs show data from 4 REG leads: left and right fronto-mastoid (FM), left and right occcipito-mastoid (OM) for sitting and supine positions. The graphs show confidence intervals with means represented by circle-shaped points, and medians depicted as rhomb-shaped points. Additionally, points and intervals are highlighted by different colors to distinguish between first sitting (oSA) and supine (oHA) positions and second sitting (oSB) and supine (oHB) positions. A one-way repeated measures ANOVA and a nonparametric Friedman test summaries for statistically significant results: left FM (F (1.565, 48.53) = 10.37, p = 0.0005), left OM (Friedman statistic = 14.36, p = 0.0025). “*” –p < 0.05, “**” –p < 0.01, “***” –p < 0.001. [file peerj-13-20233-s024.pdf]

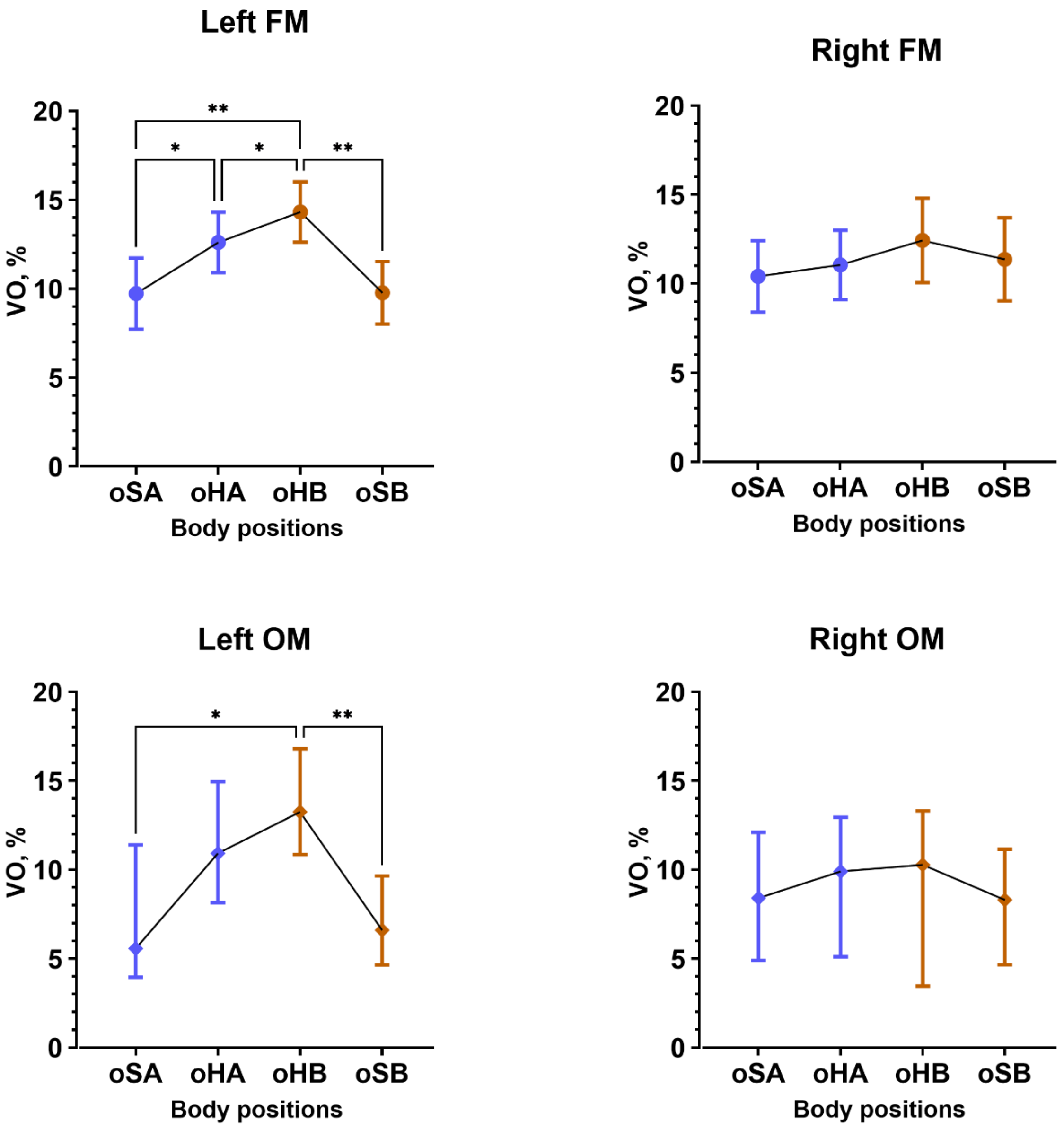

Supplemental Figure 17. Postural changes of VO among all participants during Test 2 (n = 32). The graphs show data from 4 REG leads: left and right fronto-mastoid (FM), left and right occcipito-mastoid (OM) for sitting and supine positions. The graphs show confidence intervals with means represented by circle-shaped points, and medians depicted as rhomb-shaped points. Additionally, points and intervals are highlighted by different colors to distinguish between first sitting (oSA) and supine (oHA) positions and second sitting (oSB) and supine (oHB) positions. A one-way repeated measures ANOVA and a nonparametric Friedman test summaries for statistically significant results: left FM (F (1.565, 48.53) = 10.37, p = 0.0005), left OM (*Friedman statistic* = 14.36, p = 0.0025). "*" – p < 0.05, "**" – p < 0.01, "***" – p < 0.001.
