# Supplementary material for: The role of cerebral blood flow volume in cortical inhibition during postural changes
Source: PeerJ. 2025 Oct 27;13:e20233. doi: 10.7717/peerj.20233 (PMC12574591; doi:10.7717/peerj.20233)
Supplement: Supplemental Information 25 — The graphs show data from 4 REG leads: left and right fronto-mastoid (FM), left and right occcipito-mastoid (OM) for two sitting positions (SA and SB). Black boxplots include values of male participants (m), and red boxplots contain values of female participants (f). Pairs of boxplots were analyzed separately using one-way ANOVA, i.e., SA (m) was compared only to SA (f), and SB (m) was compared only to SB (f). Outliers are shown by black and blue points. [file peerj-13-20233-s025.pdf]

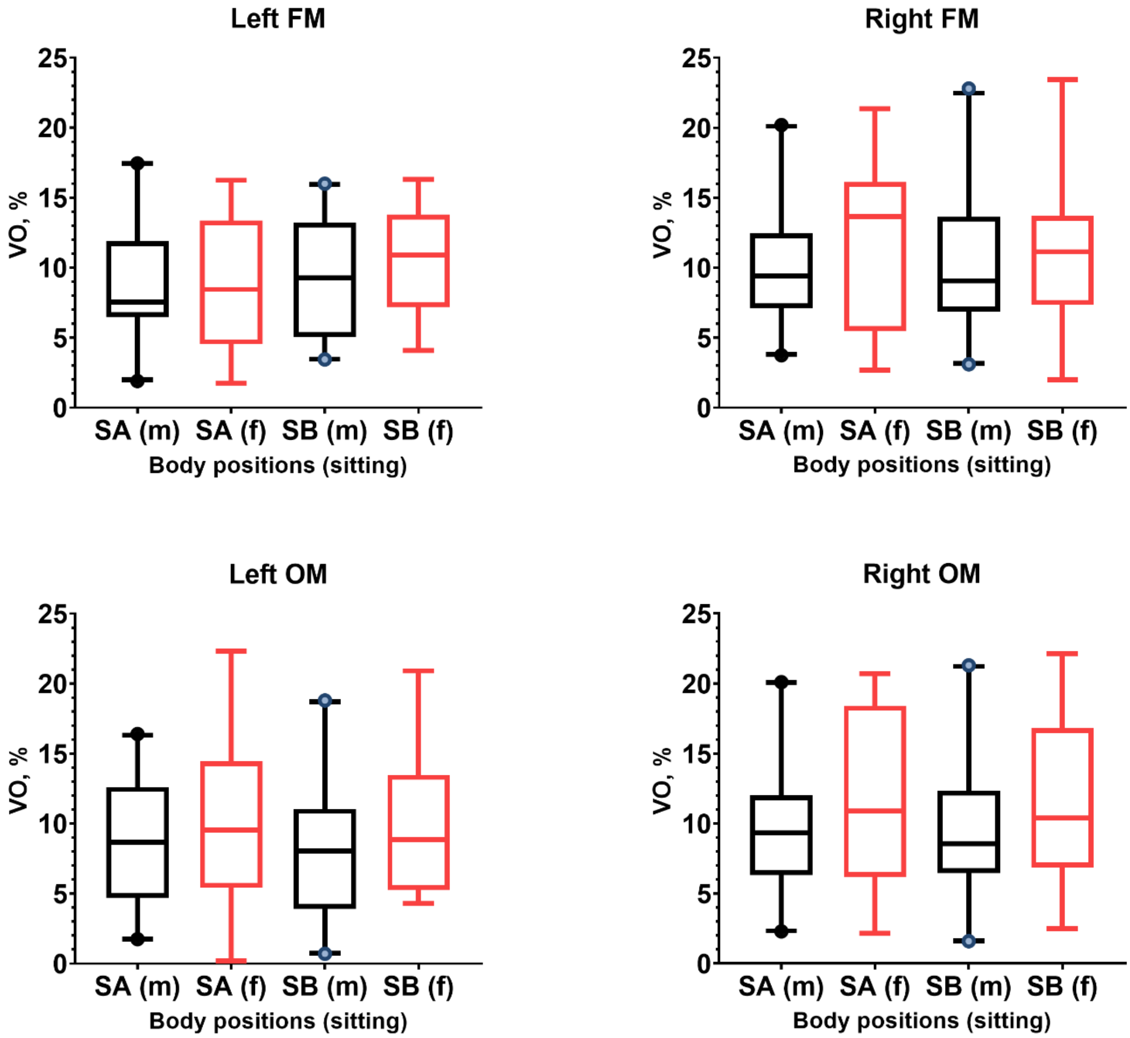

Supplemental Figure 18. Sex differences in VO during sitting positions in Test 1 (*n* = 37). The graphs show data from 4 REG leads: left and right fronto-mastoid (FM), left and right occcipito-mastoid (OM) for two sitting positions (SA and SB). Black boxplots include values of male participants (m), and red boxplots contain values of female participants (f). Pairs of boxplots were analyzed separately using one-way ANOVA, i.e., SA (m) was compared only to SA (f), and SB (m) was compared only to SB (f). Outliers are shown by black and blue points.
