# Supplementary material for: The role of cerebral blood flow volume in cortical inhibition during postural changes
Source: PeerJ. 2025 Oct 27;13:e20233. doi: 10.7717/peerj.20233 (PMC12574591; doi:10.7717/peerj.20233)
Supplement: Supplemental Information 26 — The graphs show data from 4 REG leads: left and right fronto-mastoid (FM), left and right occcipito-mastoid (OM) for the first and last 2 min of supine position (HA and HB). Black boxplots include values of male participants (m), and red boxplots contain values of female participants (f). Pairs of boxplots were analyzed separately using one-way ANOVA, i.e., HA (m) was compared only to HA (f), and HB (m) was compared only to HB (f). Outliers are shown by black and blue points. [file peerj-13-20233-s026.pdf]

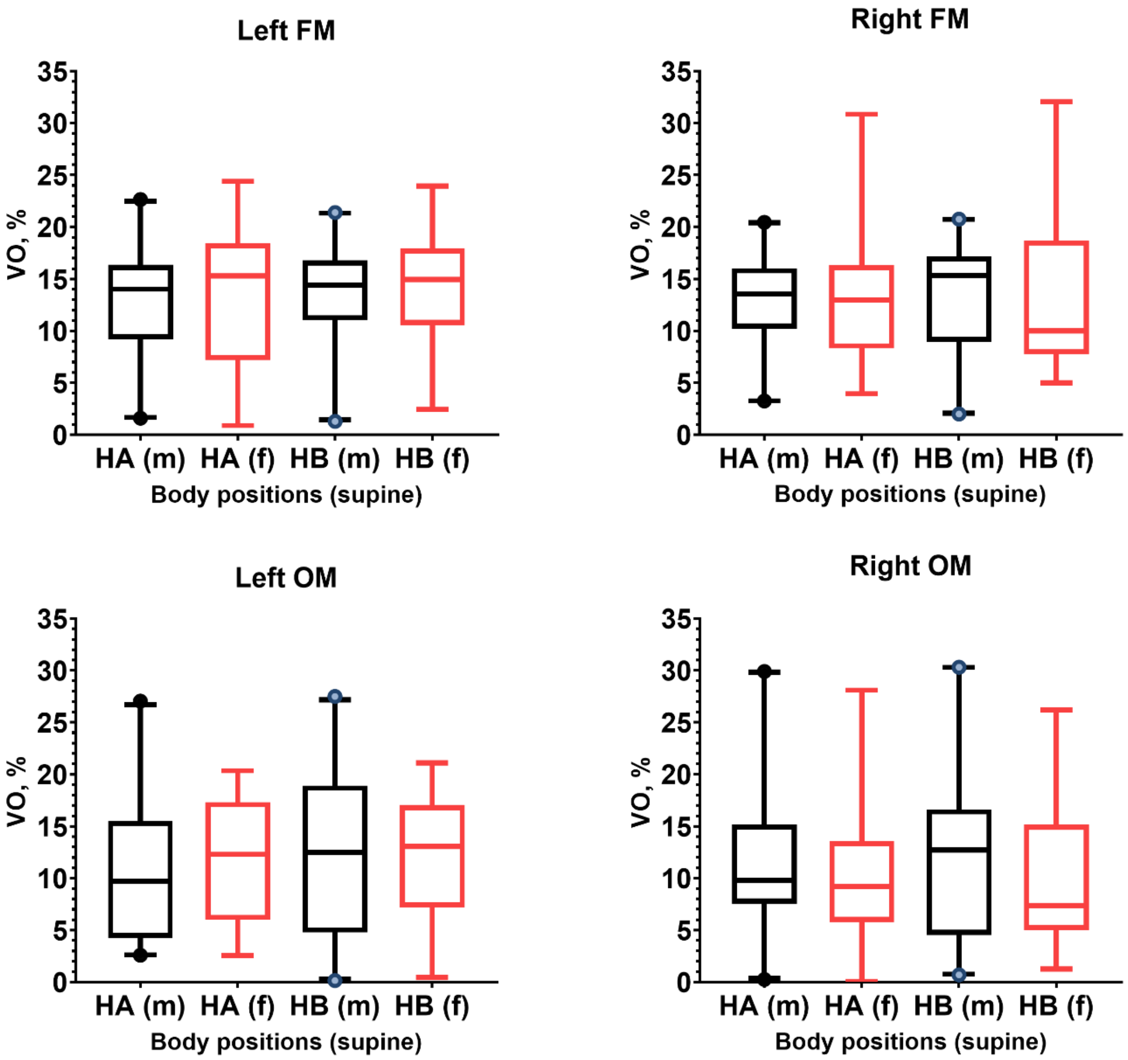

Supplemental Figure 19. Sex differences in VO during supine positions in Test 1 (*n* = 37). The graphs show data from 4 REG leads: left and right fronto-mastoid (FM), left and right occcipito-mastoid (OM) for the first and last 2 minutes of supine position (HA and HB). Black boxplots include values of male participants (m), and red boxplots contain values of female participants (f). Pairs of boxplots were analyzed separately using one-way ANOVA, i.e., HA (m) was compared only to HA (f), and HB (m) was compared only to HB (f). Outliers are shown by black and blue points.
