# Supplementary material for: The role of cerebral blood flow volume in cortical inhibition during postural changes
Source: PeerJ. 2025 Oct 27;13:e20233. doi: 10.7717/peerj.20233 (PMC12574591; doi:10.7717/peerj.20233)
Supplement: Supplemental Information 27 — The graphs show data from 4 REG leads: left and right fronto-mastoid (FM), left and right occcipito-mastoid (OM) for two sitting positions (oSA and oSB). Black boxplots include values of male participants (m), and red boxplots contain values of female participants (f). Pairs of boxplots were analyzed separately using one-way ANOVA, i.e., oSA (m) was compared only to oSA (f), and oSB (m) was compared only to oSB (f). A one-way ANOVA and a nonparametric Kruskal–Wallis test summaries for statistically significant results: left FM (F (3, 60) = 4.447, p = 0.0069), left OM (F (3, 60) = 3.268, p < 0.0273), right OM (F (3, 60) = 3.794, p = 0.0147). “*” –p < 0.05, “**” –p < 0,01. [file peerj-13-20233-s027.pdf]

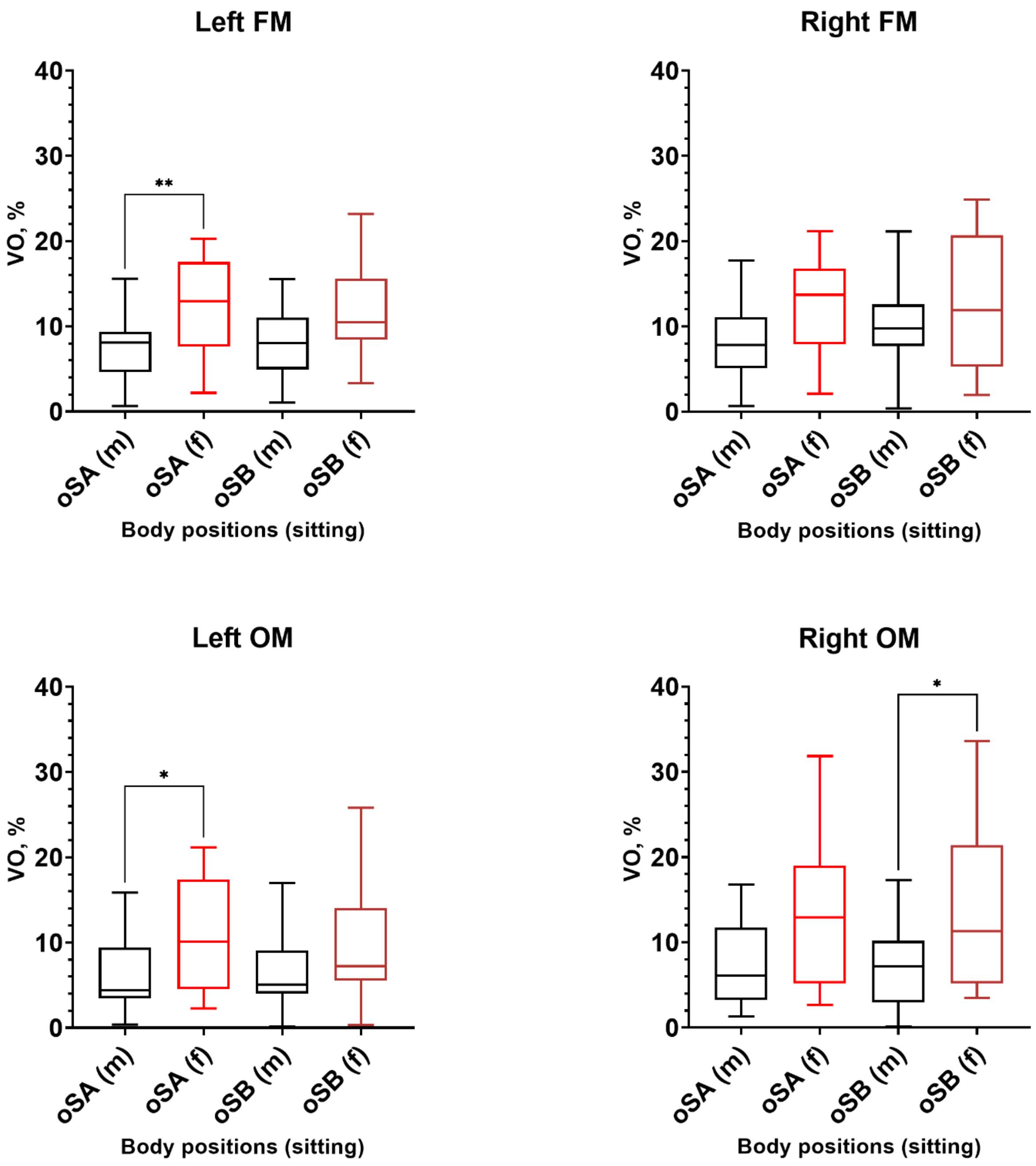

Supplemental Figure 20. Sex differences in VO during sitting positions in Test 2 (*n* = 32). The graphs show data from 4 REG leads: left and right fronto-mastoid (FM), left and right occcipito-mastoid (OM) for two sitting positions (oSA and oSB). Black boxplots include values of male participants (m), and red boxplots contain values of female participants (f). Pairs of boxplots were analyzed separately using one-way ANOVA, i.e., oSA (m) was compared only to oSA (f), and oSB (m) was compared only to oSB (f). A one-way ANOVA and a nonparametric Kruskal-Wallis test summaries for statistically significant results: left FM (F (3, 60) = 4.447, p = 0.0069), left OM (F (3, 60) = 3.268, p < 0.0273), right OM (F (3, 60) = 3.794, p = 0.0147). "*" – p < 0.05, "**" – p < 0,01.
