# Supplementary material for: The role of cerebral blood flow volume in cortical inhibition during postural changes
Source: PeerJ. 2025 Oct 27;13:e20233. doi: 10.7717/peerj.20233 (PMC12574591; doi:10.7717/peerj.20233)
Supplement: Supplemental Information 30 — The graphs show data from 4 REG leads: left and right fronto-mastoid (FM), left and right occcipito-mastoid (OM) for sitting and supine positions. The graphs show confidence intervals with medians depicted as rhomb-shaped points. Additionally, points and intervals are highlighted by different colors to distinguish between first sitting (oSA) and supine (oHA) positions and second sitting (oSB) and supine (oHB) positions. A nonparametric Friedman test summary for statistically significant results: right OM (Friedman statistic = 17.31, p = 0.0006). “*” –p < 0.05. [file peerj-13-20233-s030.pdf]

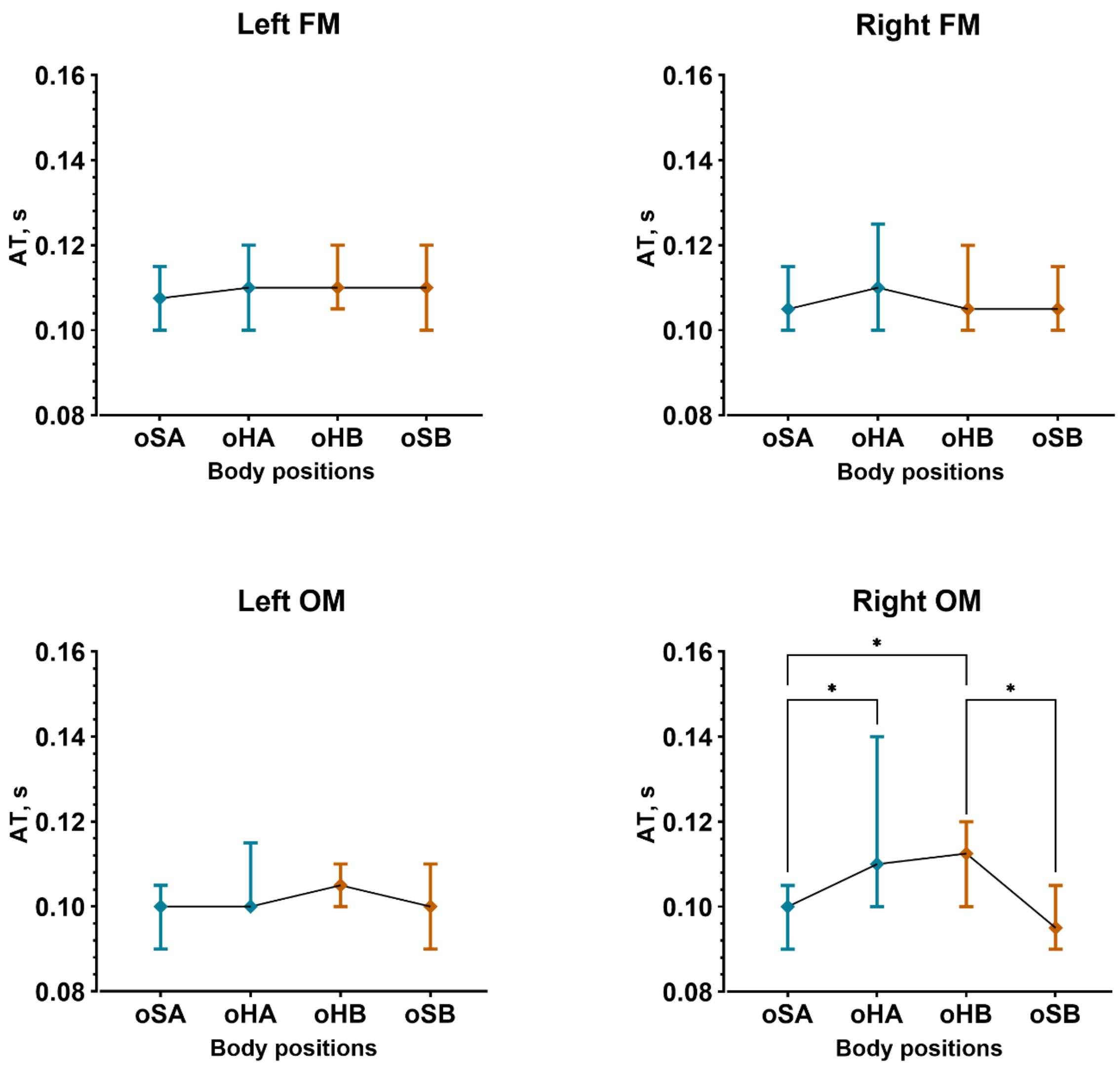

Supplemental Figure 23. Postural changes of AT among male participants during Test 2 (n = 16). The graphs show data from 4 REG leads: left and right fronto-mastoid (FM), left and right occcipito-mastoid (OM) for sitting and supine positions. The graphs show confidence intervals with medians depicted as rhomb-shaped points. Additionally, points and intervals are highlighted by different colors to distinguish between first sitting (oSA) and supine (oHA) positions and second sitting (oSB) and supine (oHB) positions. A nonparametric Friedman test summary for statistically significant results: right OM (*Friedman statistic* = 17.31, $p = 0.0006$). "*" – $p < 0.05$.
