# Supplementary material for: The role of cerebral blood flow volume in cortical inhibition during postural changes
Source: PeerJ. 2025 Oct 27;13:e20233. doi: 10.7717/peerj.20233 (PMC12574591; doi:10.7717/peerj.20233)
Supplement: Supplemental Information 32 — The graphs show data from 4 REG leads: left and right fronto-mastoid (FM), left and right occcipito-mastoid (OM) for sitting and supine positions. The graphs show confidence intervals with means depicted as circle-shaped points. Additionally, points and intervals are highlighted by different colors to distinguish between first sitting (SA) and first 2 min of supine (HA) position and second sitting (SB) and last 2 min of supine (HB) position. A one-way repeated measures ANOVA summary for statistically significant results: left FM (F (1.259, 23.91) = 61.4, p < 0.0001), right FM (F (1.384, 26.29) = 34.39, p < 0.0001), left OM (F (1.63, 30.98) = 40.61, p < 0.0001), right OM (F (1.636, 31.09) = 19.23, p < 0.0001). “*” –p < 0.05, “***” –p < 0.001, “****” –p < 0.0001. [file peerj-13-20233-s032.pdf]

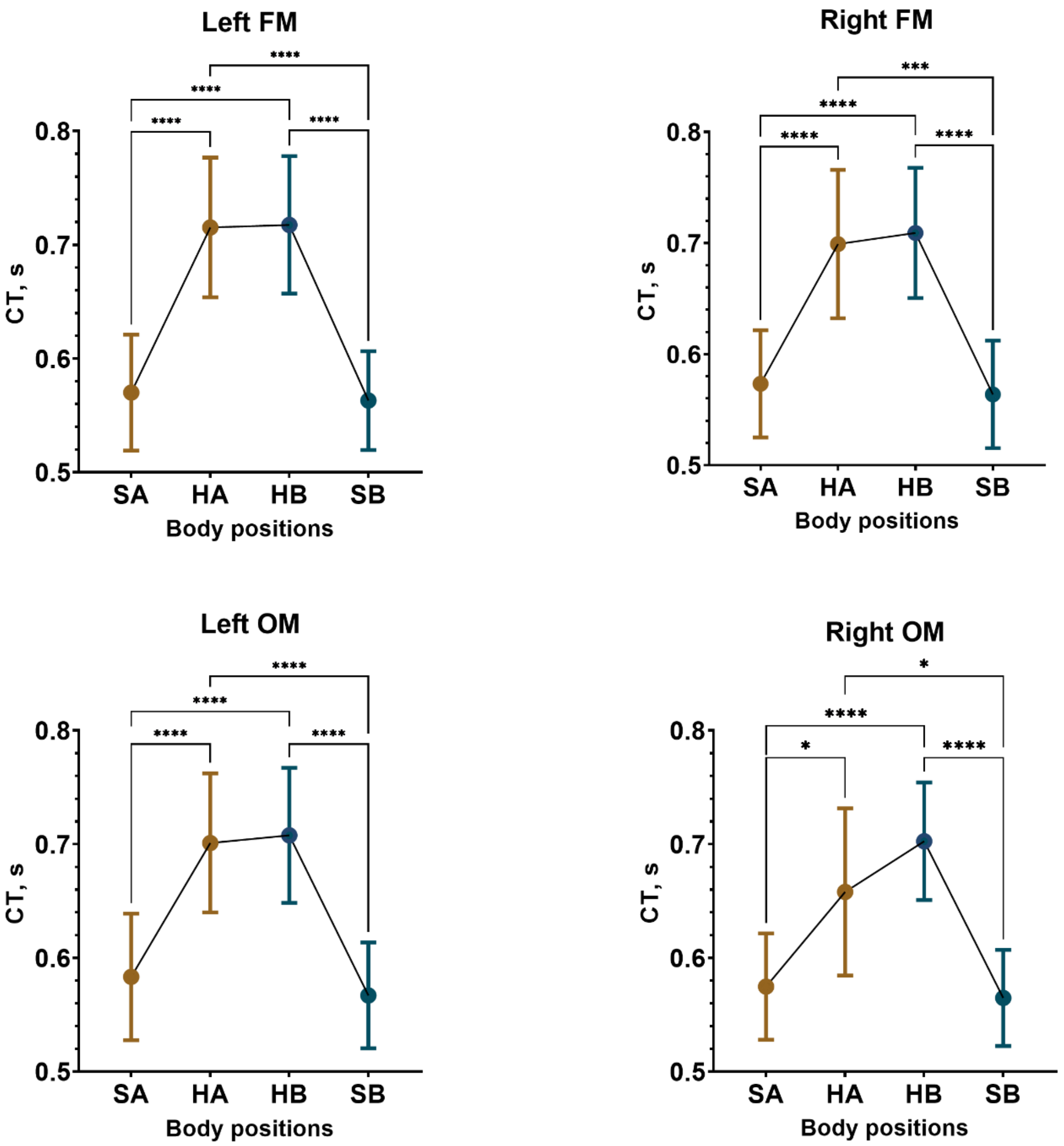

Supplemental Figure 25. Postural changes of CT among male participants during Test 1 (n = 20). The graphs show data from 4 REG leads: left and right fronto-mastoid (FM), left and right occcipito-mastoid (OM) for sitting and supine positions. The graphs show confidence intervals with means depicted as circle-shaped points. Additionally, points and intervals are highlighted by different colors to distinguish between first sitting (SA) and first 2 minutes of supine (HA) position and second sitting (SB) and last 2 minutes of supine (HB) position. A one-way repeated measures ANOVA summary for statistically significant results: left FM (F (1.259, 23.91) = 61.4, $p < 0.0001$), right FM (F (1.384, 26.29) = 34.39, $p < 0.0001$), left OM (F (1.63, 30.98) = 40.61, $p < 0.0001$), right OM (F (1.636, 31.09) = 19.23, $p < 0.0001$). "*" – $p < 0.05$, "***" – $p < 0.001$, "****" – $p < 0.0001$.
