# Supplementary material for: The role of cerebral blood flow volume in cortical inhibition during postural changes
Source: PeerJ. 2025 Oct 27;13:e20233. doi: 10.7717/peerj.20233 (PMC12574591; doi:10.7717/peerj.20233)
Supplement: Supplemental Information 33 — The graphs show data from 4 REG leads: left and right fronto-mastoid (FM), left and right occcipito-mastoid (OM) for sitting and supine positions. The graphs show confidence intervals with means represented by circle-shaped points, and medians depicted as rhomb-shaped points. Additionally, points and intervals are highlighted by different colors to distinguish between first sitting (SA) and first 2 min of supine (HA) position and second sitting (SB) and last 2 min of supine (HB) position. A nonparametric Friedman test summary for statistically significant results: left FM (Friedman statistic = 41.13, p < 0.0001), right FM (Friedman statistic = 41.61, p < 0.0001). “***” –p < 0.001, “****” –p < 0.0001. [file peerj-13-20233-s033.pdf]

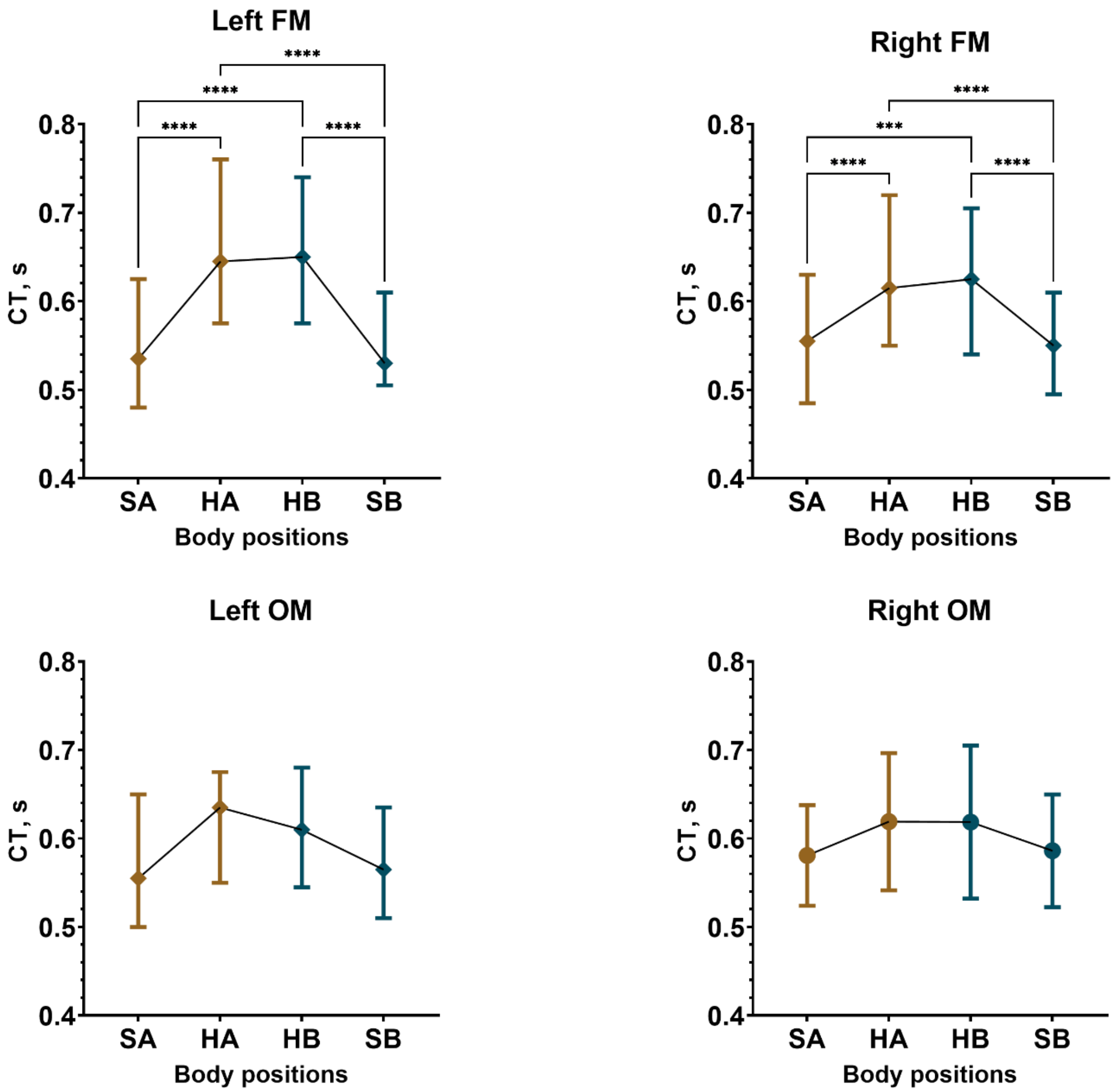

Supplemental Figure 26. Postural changes of CT among female participants during Test 1 (n = 17). The graphs show data from 4 REG leads: left and right fronto-mastoid (FM), left and right occcipito-mastoid (OM) for sitting and supine positions. The graphs show confidence intervals with means represented by circle-shaped points, and medians depicted as rhomb-shaped points. Additionally, points and intervals are highlighted by different colors to distinguish between first sitting (SA) and first 2 minutes of supine (HA) position and second sitting (SB) and last 2 minutes of supine (HB) position. A nonparametric Friedman test summary for statistically significant results: left FM (*Friedman statistic* = 41.13, $p < 0.0001$), right FM (*Friedman statistic* = 41.61, $p < 0.0001$). "***" – $p < 0.001$, "****" – $p < 0.0001$.
