# Supplementary material for: The role of cerebral blood flow volume in cortical inhibition during postural changes
Source: PeerJ. 2025 Oct 27;13:e20233. doi: 10.7717/peerj.20233 (PMC12574591; doi:10.7717/peerj.20233)
Supplement: Supplemental Information 35 — The graphs show data from 4 REG leads: left and right fronto-mastoid (FM), left and right occcipito-mastoid (OM) for sitting and supine positions. The graphs show confidence intervals with means represented by circle-shaped points. Additionally, points and intervals are highlighted by different colors to distinguish between first sitting (oSA) and supine (oHA) positions and second sitting (oSB) and supine (oHB) positions. A one-way repeated measures ANOVA summary for statistically significant results: left FM (F (1.634, 24.51) = 62.31, p < 0.0001), right FM (F (1.582, 23.73) = 54.73, p < 0.0001), left OM (F (1,.804, 27.06) = 33.33, p < 0.0001), right OM (F (1.446, 21.69) = 36.55, p < 0.0001). “*” –p < 0.05, “***” –p < 0.001, “****” –p < 0.0001. [file peerj-13-20233-s035.pdf]

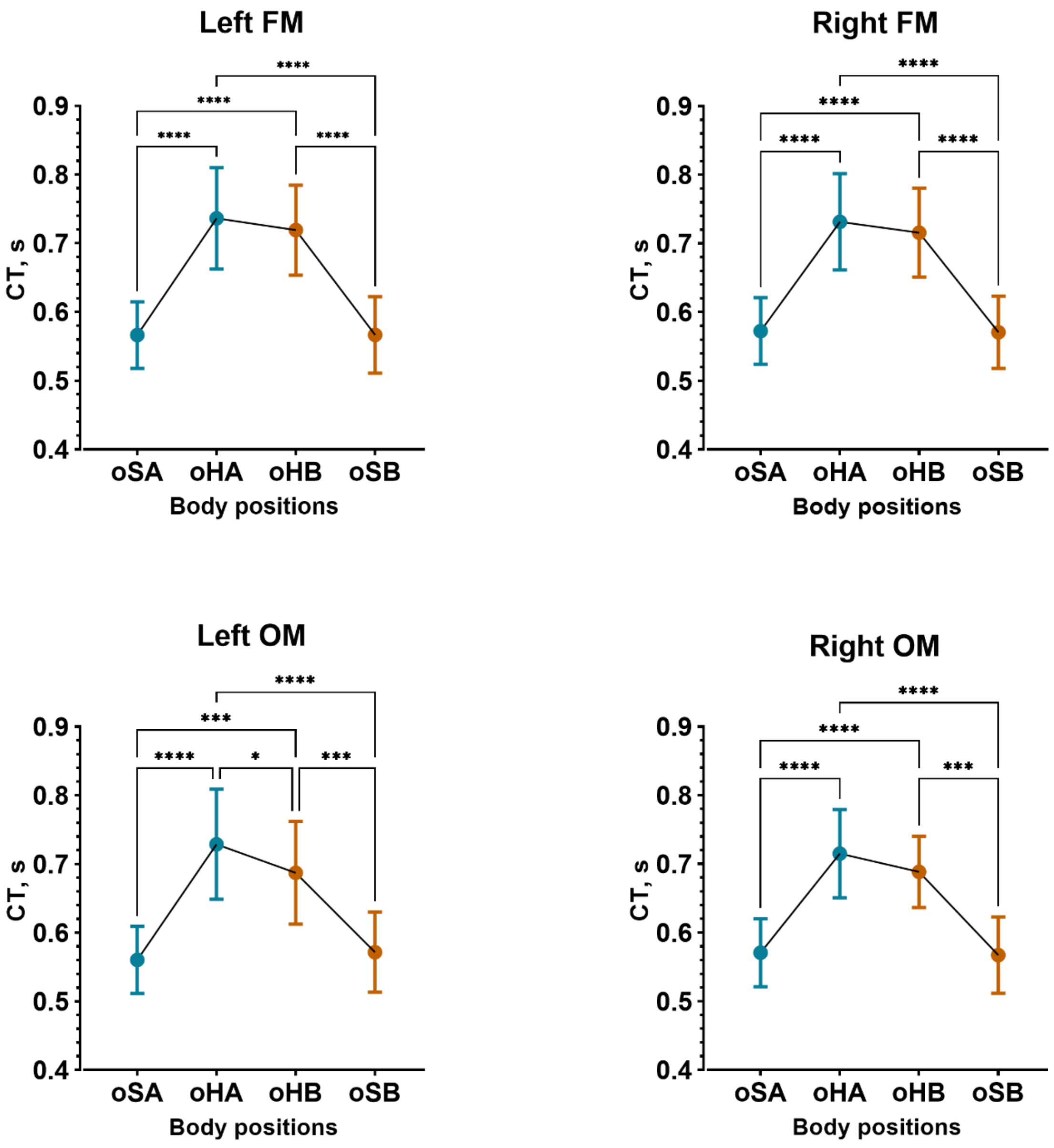

Supplemental Figure 28. Postural changes of CT among male participants during Test 2 (n = 16). The graphs show data from 4 REG leads: left and right fronto-mastoid (FM), left and right occcipito-mastoid (OM) for sitting and supine positions. The graphs show confidence intervals with means represented by circle-shaped points. Additionally, points and intervals are highlighted by different colors to distinguish between first sitting (oSA) and supine (oHA) positions and second sitting (oSB) and supine (oHB) positions. A one-way repeated measures ANOVA summary for statistically significant results: left FM (F (1.634, 24.51) = 62.31, $p < 0.0001$), right FM (F (1.582, 23.73) = 54.73, $p < 0.0001$), left OM (F (1,.804, 27.06) = 33.33, $p < 0.0001$), right OM (F (1.446, 21.69) = 36.55, $p < 0.0001$). "*" – $p < 0.05$, "***" – $p < 0.001$, "****" – $p < 0.0001$.
