# Supplementary material for: The role of cerebral blood flow volume in cortical inhibition during postural changes
Source: PeerJ. 2025 Oct 27;13:e20233. doi: 10.7717/peerj.20233 (PMC12574591; doi:10.7717/peerj.20233)
Supplement: Supplemental Information 36 — The graphs show data from 4 REG leads: left and right fronto-mastoid (FM), left and right occcipito-mastoid (OM) for sitting and supine positions. The graphs show confidence intervals with means represented by circle-shaped points, and medians depicted as rhomb-shaped points. Additionally, points and intervals are highlighted by different colors to distinguish between first sitting (oSA) and supine (oHA) positions and second sitting (oSB) and supine (oHB) positions. A one-way repeated measures ANOVA and a nonparametric Friedman test summaries for statistically significant results : left FM (Friedman statistic = 27.95, p < 0.0001), right FM (F (1.969, 29.54) = 4.443, p = 0.021). “*” –p < 0.05, “**” –p < 0.01, “***” –p < 0.001. [file peerj-13-20233-s036.pdf]

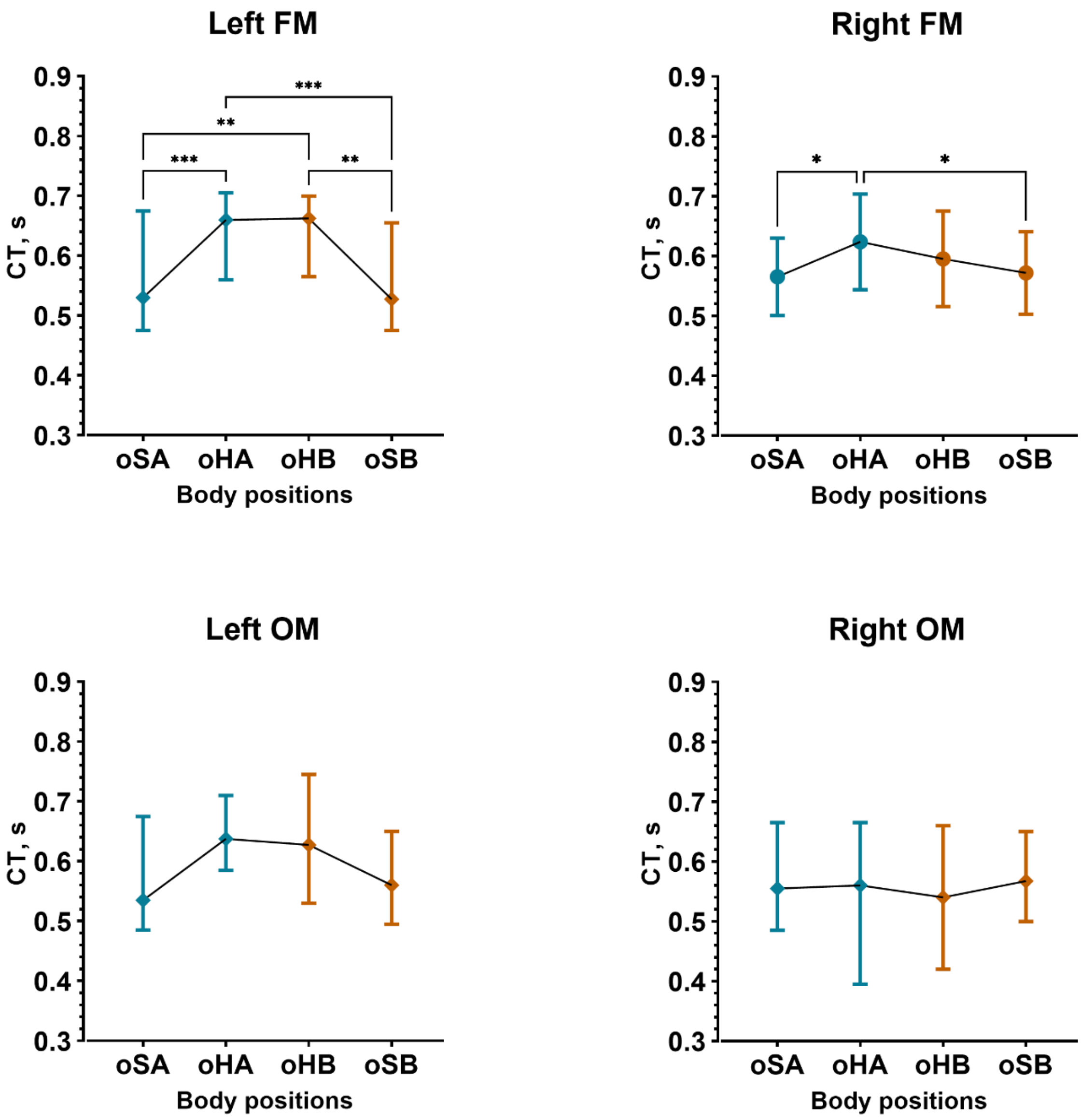

Supplemental Figure 29. Postural changes of CT among female participants during Test 2 (n = 16). The graphs show data from 4 REG leads: left and right fronto-mastoid (FM), left and right occcipito-mastoid (OM) for sitting and supine positions. The graphs show confidence intervals with means represented by circle-shaped points, and medians depicted as rhomb-shaped points. Additionally, points and intervals are highlighted by different colors to distinguish between first sitting (oSA) and supine (oHA) positions and second sitting (oSB) and supine (oHB) positions. A one-way repeated measures ANOVA and a nonparametric Friedman test summaries for statistically significant results: left FM (*Friedman statistic* = 27.95, $p < 0.0001$), right FM (F (1.969, 29.54) = 4.443, $p = 0.021$). "*" – $p < 0.05$, "**" – $p < 0.01$, "***" – $p < 0.001$.
