# Supplementary material for: The role of cerebral blood flow volume in cortical inhibition during postural changes
Source: PeerJ. 2025 Oct 27;13:e20233. doi: 10.7717/peerj.20233 (PMC12574591; doi:10.7717/peerj.20233)
Supplement: Supplemental Information 37 — The graphs show data from 4 REG leads: left and right fronto-mastoid (FM), left and right occcipito-mastoid (OM) for sitting and supine positions. The graphs show confidence intervals with means represented by circle-shaped points. Additionally, points and intervals are highlighted by different colors to distinguish between first sitting (oSA) and supine (oHA) positions and second sitting (oSB) and supine (oHB) positions. A one-way repeated measures ANOVA summary for statistically significant results: left FM (F (1.665, 49.94) = 67.29, p < 0.0001), right FM (F (1.553, 46.59) = 28.32, p < 0.0001), left OM (F (1.658, 49.74) = 33.86, p < 0.0001), right OM (F (1.165, 33.77) = 4.148, p = 0.044). “***” –p < 0.001, “****” –p < 0.0001. [file peerj-13-20233-s037.pdf]

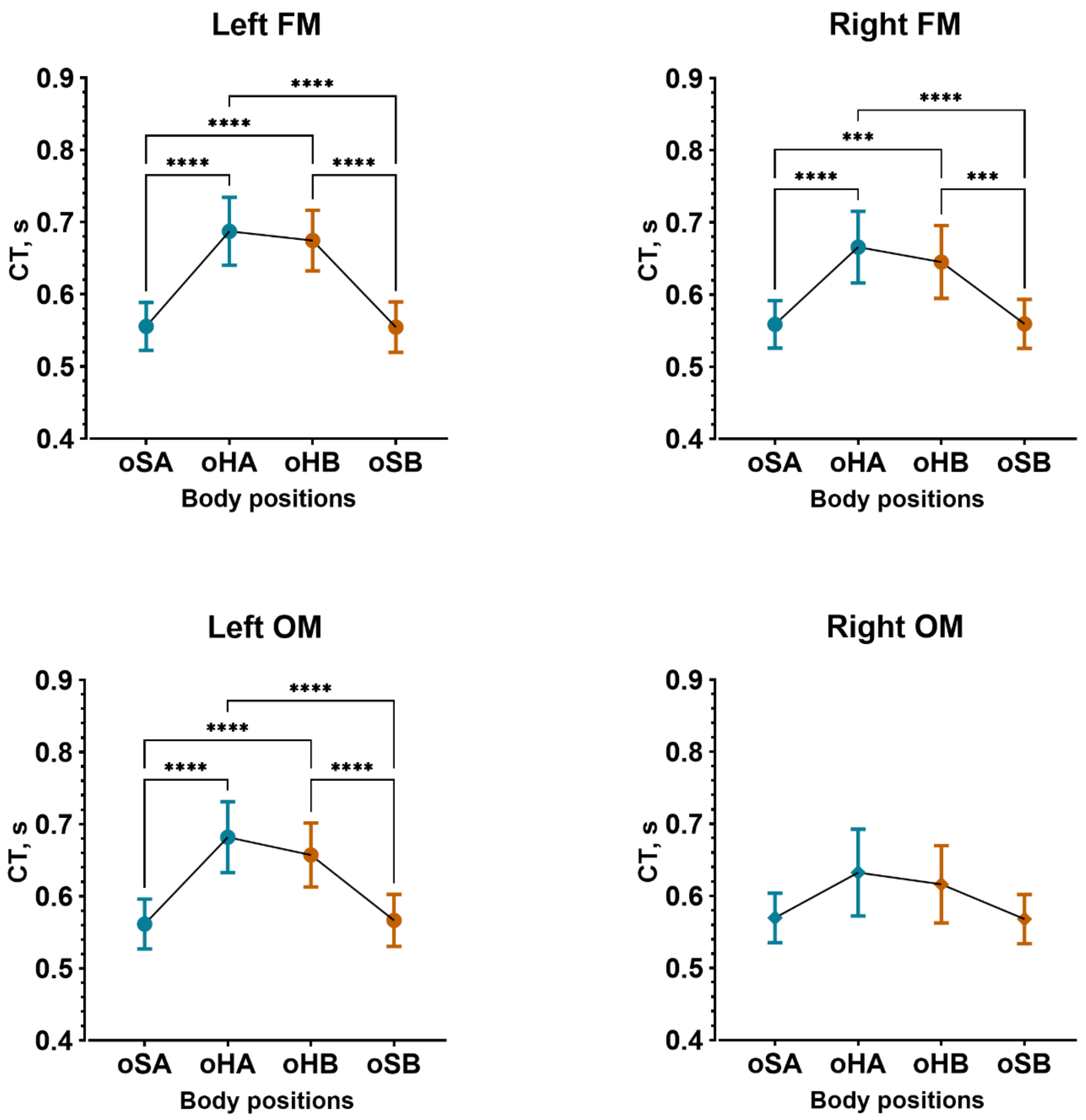

Supplemental Figure 30. Postural changes of CT among all participants during Test 2 (n = 32). The graphs show data from 4 REG leads: left and right fronto-mastoid (FM), left and right occcipito-mastoid (OM) for sitting and supine positions. The graphs show confidence intervals with means represented by circle-shaped points. Additionally, points and intervals are highlighted by different colors to distinguish between first sitting (oSA) and supine (oHA) positions and second sitting (oSB) and supine (oHB) positions. A one-way repeated measures ANOVA summary for statistically significant results: left FM (F (1.665, 49.94) = 67.29, $p <$ 0.0001), right FM (F (1.553, 46.59) = 28.32, $p <$ 0.0001), left OM (F (1.658, 49.74) = 33.86, $p <$ 0.0001), right OM (F (1.165, 33.77) = 4.148, p = 0.044). "***" – $p <$ 0.001, "****" – $p <$ 0.0001.
