# Supplementary material for: The role of cerebral blood flow volume in cortical inhibition during postural changes
Source: PeerJ. 2025 Oct 27;13:e20233. doi: 10.7717/peerj.20233 (PMC12574591; doi:10.7717/peerj.20233)
Supplement: Supplemental Information 38 — The graphs show confidence intervals with means represented by circle-shaped points, and medians depicted as rhomb-shaped points. Additionally, points and intervals are highlighted by different colors to distinguish between first sitting (SA) and first 2 min of supine (HA) position and second sitting (SB) and last 2 min of supine (HB) position. A one-way repeated measures ANOVA and a nonparametric Friedman test summaries for statistically significant results: F3 (F (1.840, 33.12) = 13.37, p < 0.0001), F4 (F (1.606, 28.92) = 9.69, p = 0.0012), F7 (Friedman statistic = 26.81, p < 0.0001), F8 (Friedman statistic = 26.59, p < 0.0001). “*” –p < 0.05, “**” –p < 0.01, “****” –p < 0.0001. [file peerj-13-20233-s038.pdf]

Postural changes in spectral power of alpha waves

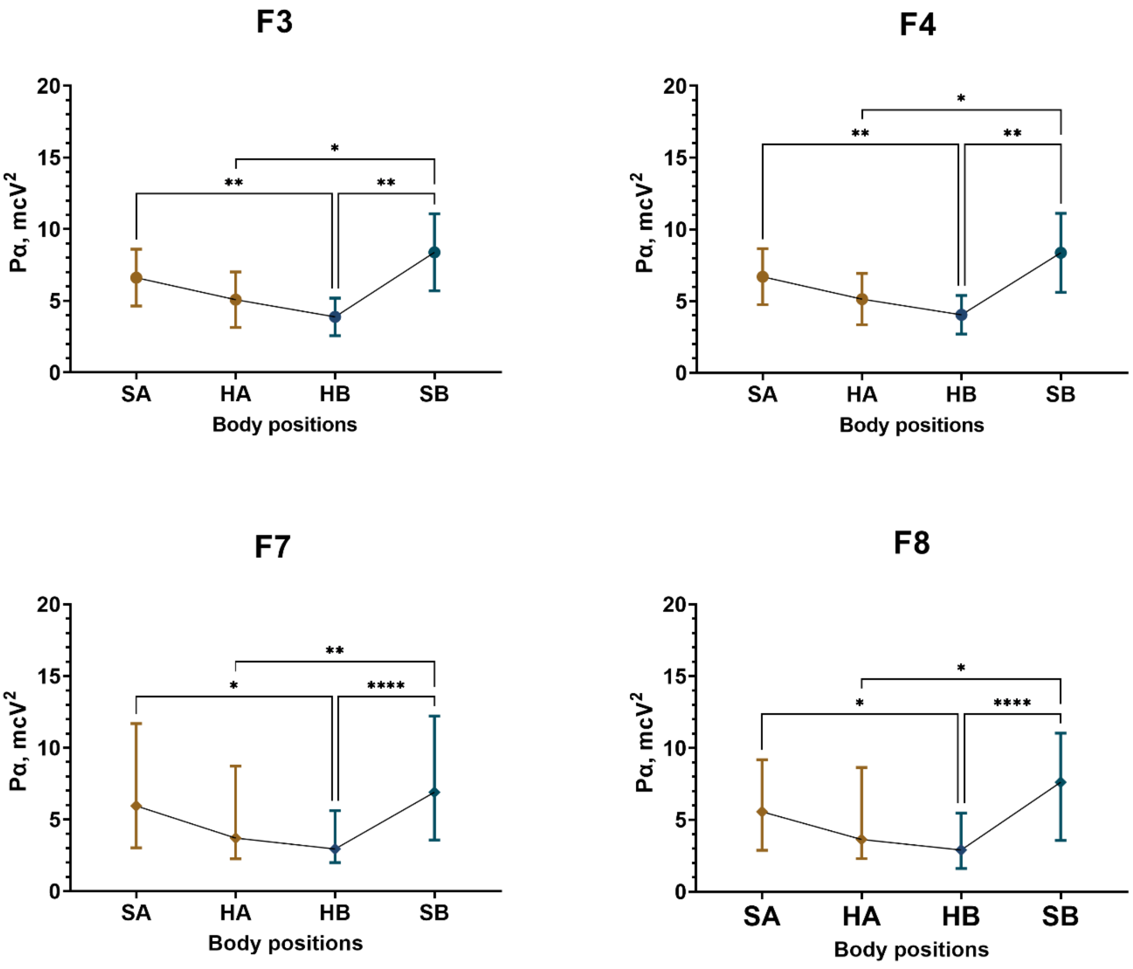

Supplemental Figure 31. Postural changes of alpha spectral power (Pα) calculated for F3, F4, F7 and F8 electrodes among male participants during Test 1 (n = 19). The graphs show confidence intervals with means represented by circle-shaped points, and medians depicted as rhomb-shaped points. Additionally, points and intervals are highlighted by different colors to distinguish between first sitting (SA) and first 2 minutes of supine (HA) position and second sitting (SB) and last 2 minutes of supine (HB) position. A one-way repeated measures ANOVA and a nonparametric Friedman test summaries for statistically significant results: F3 (F (1.840, 33.12) = 13.37, $p < 0.0001$), F4 (F (1.606, 28.92) = 9.69, $p = 0.0012$), F7 (*Friedman statistic* = 26.81, $p < 0.0001$), F8 (*Friedman statistic* = 26.59, $p < 0.0001$). "*" – p < 0.05, "**" – p < 0.01, "****" – $p < 0.0001$.
