# Supplementary material for: The role of cerebral blood flow volume in cortical inhibition during postural changes
Source: PeerJ. 2025 Oct 27;13:e20233. doi: 10.7717/peerj.20233 (PMC12574591; doi:10.7717/peerj.20233)
Supplement: Supplemental Information 40 — The graphs show confidence intervals with means represented by circle-shaped points, and medians depicted as rhomb-shaped points. Additionally, points and intervals are highlighted by different colors to distinguish between first sitting (SA) and first 2 min of supine (HA) position and second sitting (SB) and last 2 min of supine (HB) position. A one-way repeated measures ANOVA and a nonparametric Friedman test summaries for statistically significant results: F3 (F (2.057, 63.76) = 14.65, p < 0.0001), F4 (F (2.016, 62.49) = 11.43, p < 0.0001), F7 (F (2.11, 63.31) = 10.47, p < 0.0001), F8 (Friedman statistic = 28.39, p < 0.0001). “*” –p < 0.05, “**” –p < 0.01, “***” –p < 0.001, “****” –p < 0.0001. [file peerj-13-20233-s040.pdf]

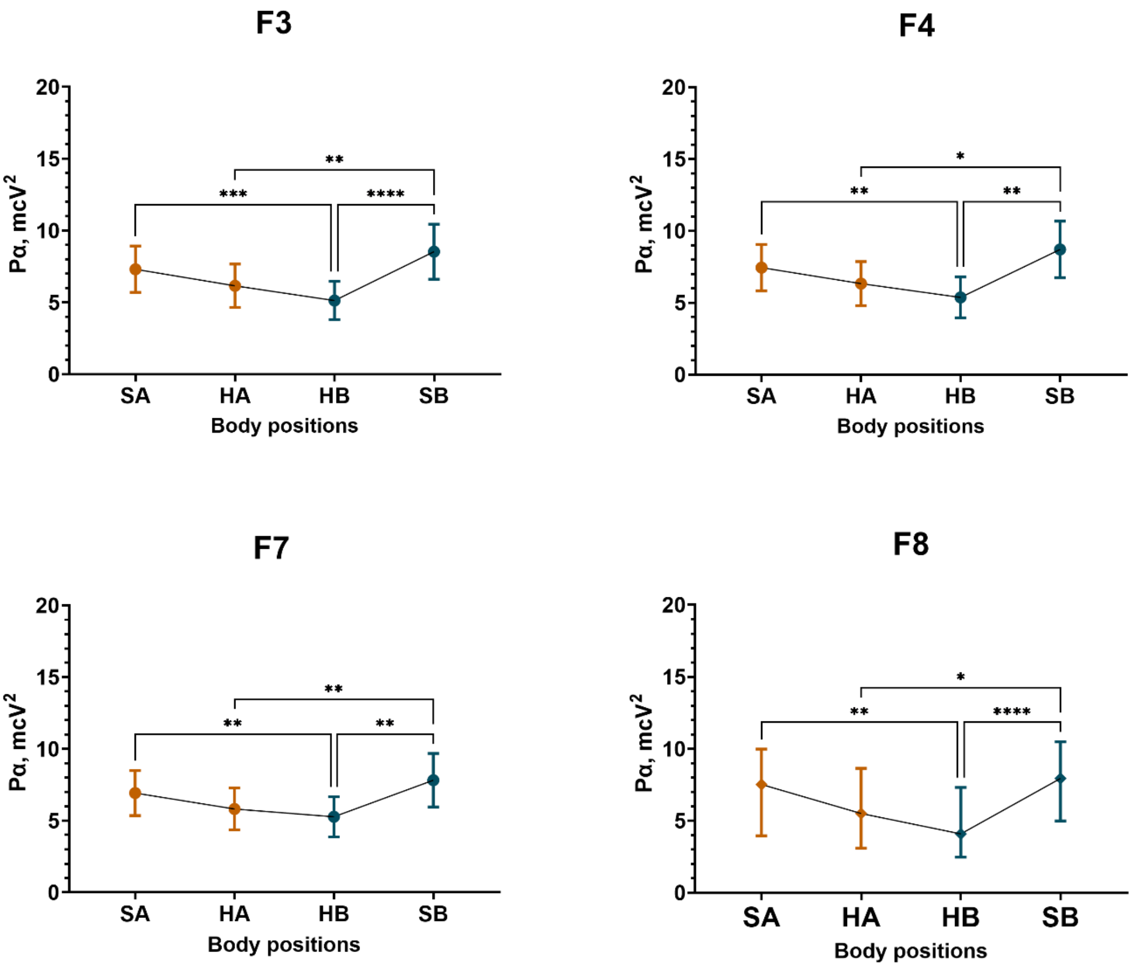

Supplemental Figure 33. Postural changes of alpha spectral power (Pα) calculated for F3, F4, F7 and F8 electrodes among all participants during Test 1 (n = 33). The graphs show confidence intervals with means represented by circle-shaped points, and medians depicted as rhomb-shaped points. Additionally, points and intervals are highlighted by different colors to distinguish between first sitting (SA) and first 2 minutes of supine (HA) position and second sitting (SB) and last 2 minutes of supine (HB) position. A one-way repeated measures ANOVA and a nonparametric Friedman test summaries for statistically significant results: F3 (F (2.057, 63.76) = 14.65, $p < 0.0001$), F4 (F (2.016, 62.49) = 11.43, $p < 0.0001$), F7 (F (2.11, 63.31) = 10.47, $p < 0.0001$), F8 (*Friedman statistic* = 28.39, $p < 0.0001$). "*" – p < 0.05, "**" – p < 0.01, "***" – $p < 0.001$, "****" – $p < 0.0001$.
