# Supplementary material for: The role of cerebral blood flow volume in cortical inhibition during postural changes
Source: PeerJ. 2025 Oct 27;13:e20233. doi: 10.7717/peerj.20233 (PMC12574591; doi:10.7717/peerj.20233)
Supplement: Supplemental Information 42 — The graphs show confidence intervals with medians represented by rhomb-shaped points. Additionally, points and intervals are highlighted by different colors to distinguish between first sitting (SA) and first 2 min of supine (HA) position and second sitting (SB) and last 2 min of supine (HB) position. A nonparametric Friedman test summary for statistically significant results: C4 (Friedman statistic = 13.13, p = 0.0044), T3 (Friedman statistic = 10.58, p = 0.0143), T4 (Friedman statistic = 10.95, p = 0.012). “*” –p < 0.05, “**” –p < 0.01. [file peerj-13-20233-s042.pdf]

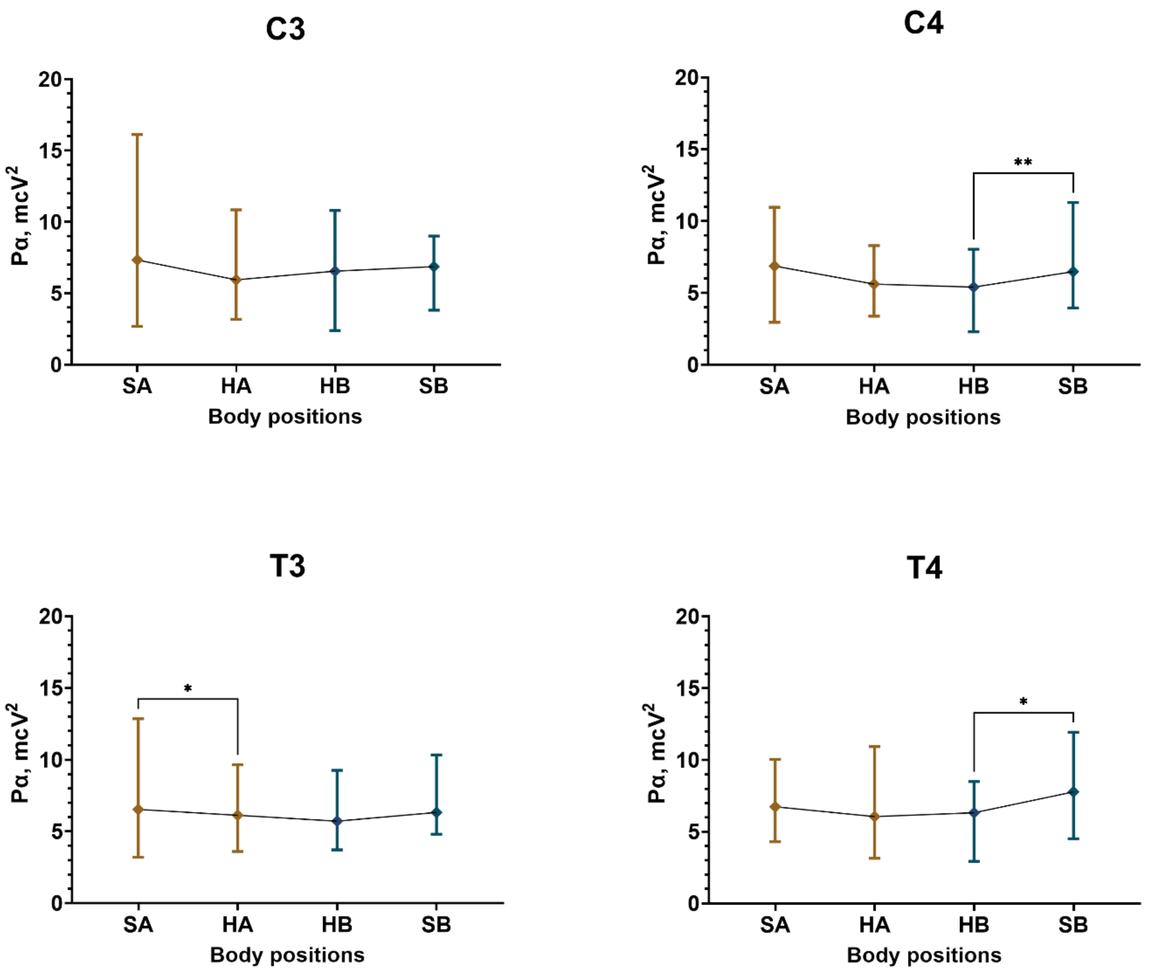

Supplemental Figure 35. Postural changes of alpha spectral power calculated for C3, C4, T3 and T4 electrodes among female participants during Test 1 (n = 16). The graphs show confidence intervals with medians represented by rhomb-shaped points. Additionally, points and intervals are highlighted by different colors to distinguish between first sitting (SA) and first 2 minutes of supine (HA) position and second sitting (SB) and last 2 minutes of supine (HB) position. A nonparametric Friedman test summary for statistically significant results: C4 (*Friedman statistic* = 13.13, $p = 0.0044$), T3 (*Friedman statistic* = 10.58, $p = 0.0143$), T4 (*Friedman statistic* = 10.95, $p = 0.012$). “*” – $p < 0.05$, “**” – $p < 0.01$.
