# Supplementary material for: The role of cerebral blood flow volume in cortical inhibition during postural changes
Source: PeerJ. 2025 Oct 27;13:e20233. doi: 10.7717/peerj.20233 (PMC12574591; doi:10.7717/peerj.20233)
Supplement: Supplemental Information 43 — The graphs show confidence intervals with means represented by circle-shaped points, and medians depicted as rhomb-shaped points. Additionally, points and intervals are highlighted by different colors to distinguish between first sitting (SA) and first 2 min of supine (HA) position and second sitting (SB) and last 2 min of supine (HB) position. A one-way repeated measures ANOVA and a nonparametric Friedman test summaries for statistically significant results: C3 (Friedman statistic = 30.38, p < 0.0001), C4 (F (2.196, 65.89) = 13.24, p < 0.0001), T3 (F (2.083, 64.57) = 9.932, p = 0.0001), T4 (F (2.453, 76.04) = 13.9, p < 0.0001). “*” –p < 0.05, “**” –p < 0.01, “***” –p < 0.001, “****” –p < 0.0001. [file peerj-13-20233-s043.pdf]

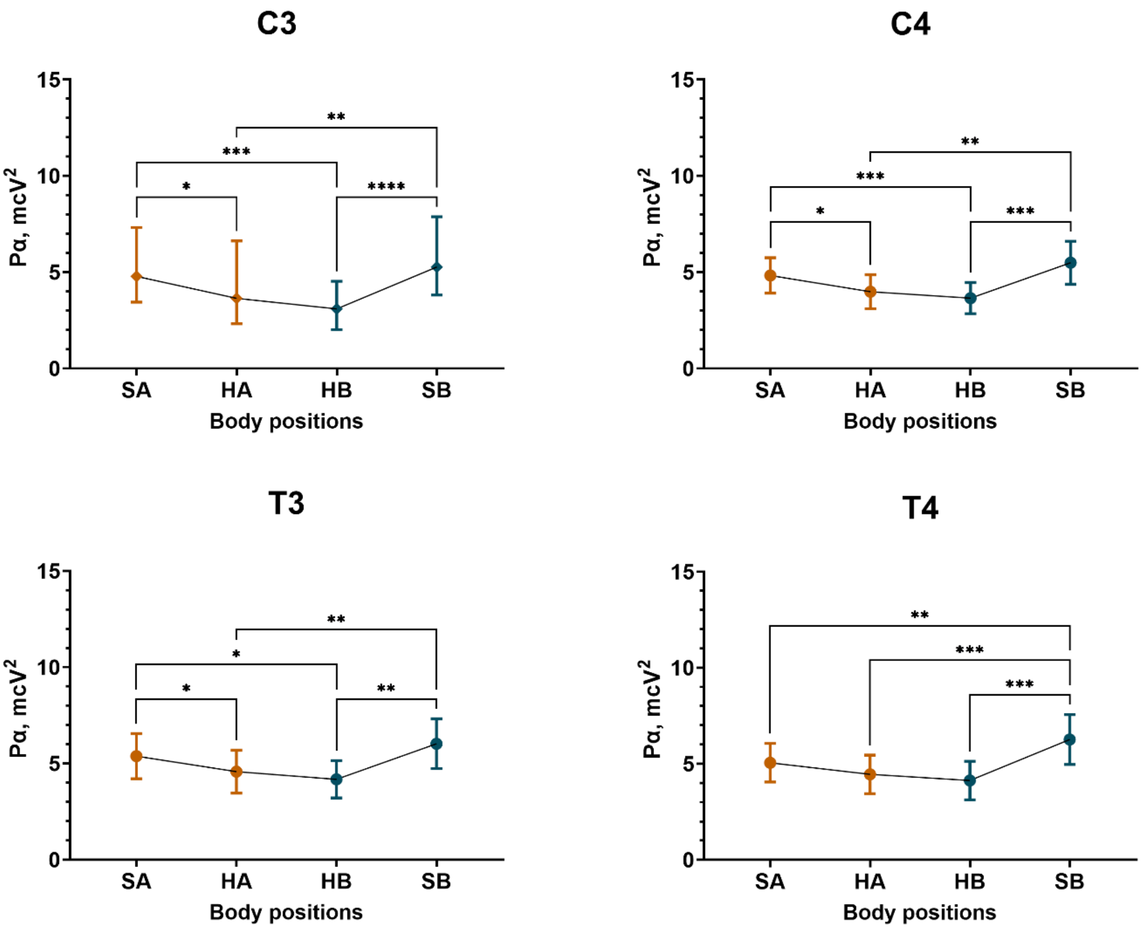

Supplemental Figure 36. Postural changes of alpha spectral power (Pα) calculated for C3, C4, T3 and T4 electrodes among all participants during Test 1 (n = 35). The graphs show confidence intervals with means represented by circle-shaped points, and medians depicted as rhomb-shaped points. Additionally, points and intervals are highlighted by different colors to distinguish between first sitting (SA) and first 2 minutes of supine (HA) position and second sitting (SB) and last 2 minutes of supine (HB) position. A one-way repeated measures ANOVA and a nonparametric Friedman test summaries for statistically significant results: C3 (Friedman statistic = 30.38, $p < 0.0001$), C4 (F (2.196, 65.89) = 13.24, $p < 0.0001$), T3 (F (2.083, 64.57) = 9.932, $p = 0.0001$), T4 (F (2.453, 76.04) = 13.9, $p < 0.0001$). "*" – $p < 0.05$, "**" – $p < 0.01$, "***" – $p < 0.001$, "****" – $p < 0.0001$.
