# Supplementary material for: The role of cerebral blood flow volume in cortical inhibition during postural changes
Source: PeerJ. 2025 Oct 27;13:e20233. doi: 10.7717/peerj.20233 (PMC12574591; doi:10.7717/peerj.20233)
Supplement: Supplemental Information 44 — The graphs show confidence intervals with means represented by circle-shaped points, and medians depicted as rhomb-shaped points. Additionally, points and intervals are highlighted by different colors to distinguish between first sitting (SA) and first 2 min of supine (HA) position and second sitting (SB) and last 2 min of supine (HB) position. A nonparametric Friedman test summaries for statistically significant results: P3 (Friedman statistic = 24.79, p < 0.0001), P4 (Friedman statistic = 40.71, p < 0.0001), T5 (Friedman statistic = 16.43, p = 0.0009), T6 (Friedman statistic = 28.2, p < 0.0001). “*” –p < 0.05, “**” –p < 0.01, “***” –p < 0.001, “****” –p < 0.0001. [file peerj-13-20233-s044.pdf]

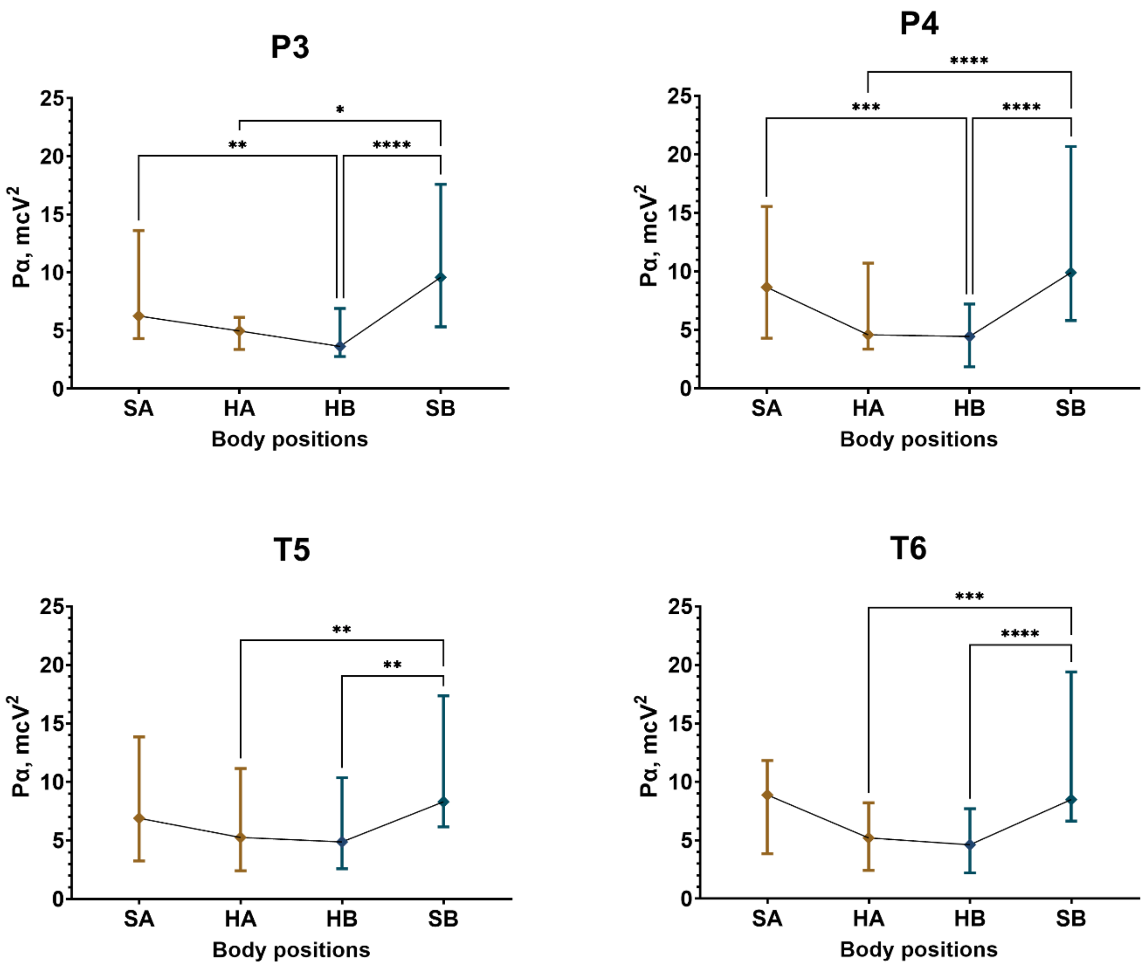

Supplementary Figure 37. Postural changes of alpha spectral power (Pα) calculated for P3, P4, T5 and T6 electrodes among male participants during Test 1 (n = 19). The graphs show confidence intervals with means represented by circle-shaped points, and medians depicted as rhomb-shaped points. Additionally, points and intervals are highlighted by different colors to distinguish between first sitting (SA) and first 2 minutes of supine (HA) position and second sitting (SB) and last 2 minutes of supine (HB) position. A nonparametric Friedman test summaries for statistically significant results: P3 (*Friedman statistic* = 24.79, $p < 0.0001$), P4 (*Friedman statistic* = 40.71, $p < 0.0001$), T5 (*Friedman statistic* = 16.43, $p = 0.0009$), T6 (*Friedman statistic* = 28.2, $p < 0.0001$). "*" – $p < 0.05$, "**" – $p < 0.01$, "***" – $p < 0.001$, "****" – $p < 0.0001$.
