# Supplementary material for: The role of cerebral blood flow volume in cortical inhibition during postural changes
Source: PeerJ. 2025 Oct 27;13:e20233. doi: 10.7717/peerj.20233 (PMC12574591; doi:10.7717/peerj.20233)
Supplement: Supplemental Information 45 — The graphs show confidence intervals with means represented by circle-shaped points. Additionally, points and intervals are highlighted by different colors to distinguish between first sitting (SA) and first 2 min of supine (HA) position and second sitting (SB) and last 2 min of supine (HB) position. [file peerj-13-20233-s045.pdf]

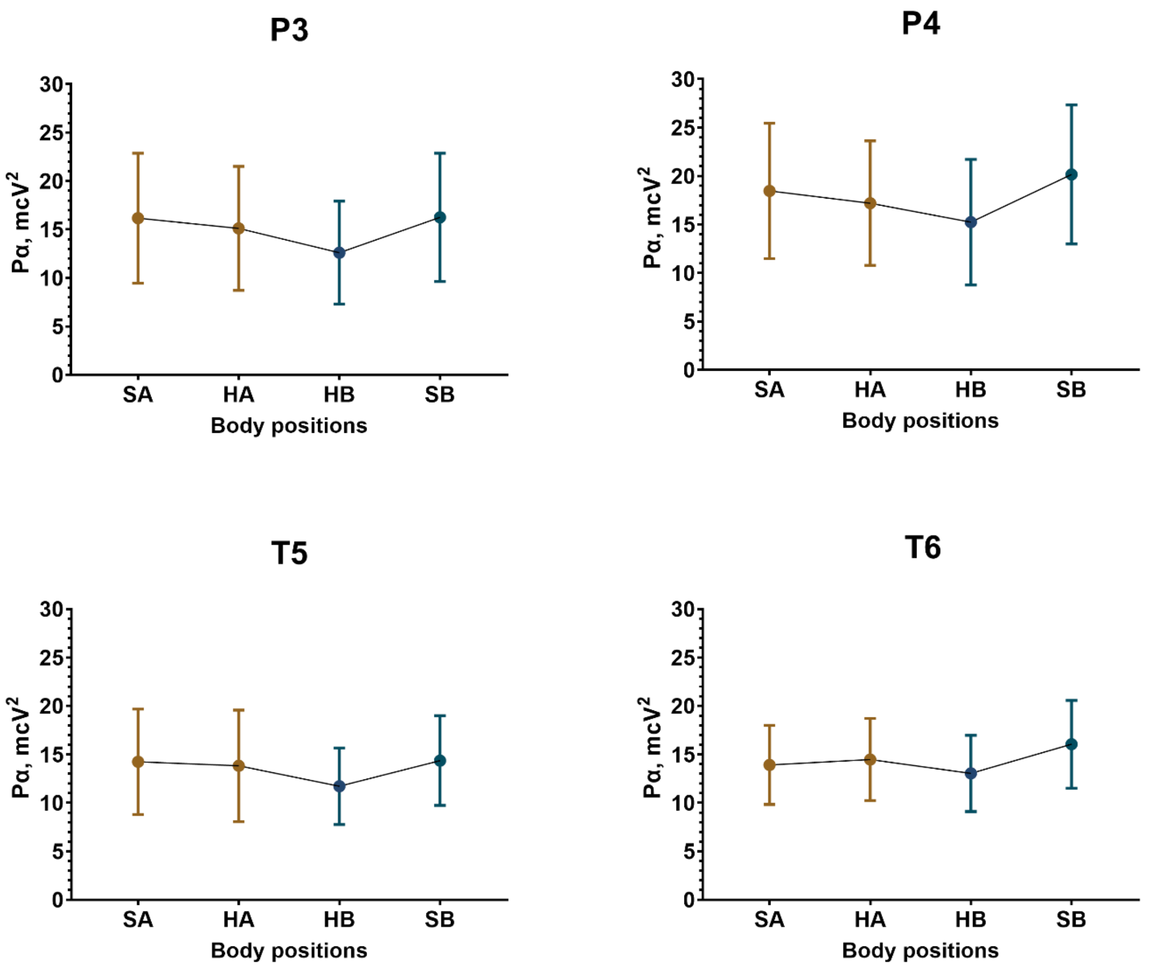

Supplementary Figure 38. Postural changes of alpha spectral power (Pα) calculated for P3, P4, T5 and T6 electrodes among female participants during Test 1 (n = 16). The graphs show confidence intervals with means represented by circle-shaped points. Additionally, points and intervals are highlighted by different colors to distinguish between first sitting (SA) and first 2 minutes of supine (HA) position and second sitting (SB) and last 2 minutes of supine (HB) position.
