# Supplementary material for: The role of cerebral blood flow volume in cortical inhibition during postural changes
Source: PeerJ. 2025 Oct 27;13:e20233. doi: 10.7717/peerj.20233 (PMC12574591; doi:10.7717/peerj.20233)
Supplement: Supplemental Information 47 — The graphs show confidence intervals with means represented by circle-shaped points, and medians depicted as rhomb-shaped points. Additionally, points and intervals are highlighted by different colors to distinguish between first sitting (SA) and first 2 min of supine (HA) position and second sitting (SB) and last 2 min of supine (HB) position. A one-way repeated measures ANOVA and a nonparametric Friedman test summaries for statistically significant results: Fz (F (1.864, 33.55) = 10.67, p = 0.0003), Cz (F (2.353, 42.35) = 11.67, p < 0.0001), Pz (Friedman statistic = 21.76, p < 0.0001). “*” –p < 0.05, “**” –p < 0.01, “***” –p < 0.001. [file peerj-13-20233-s047.pdf]

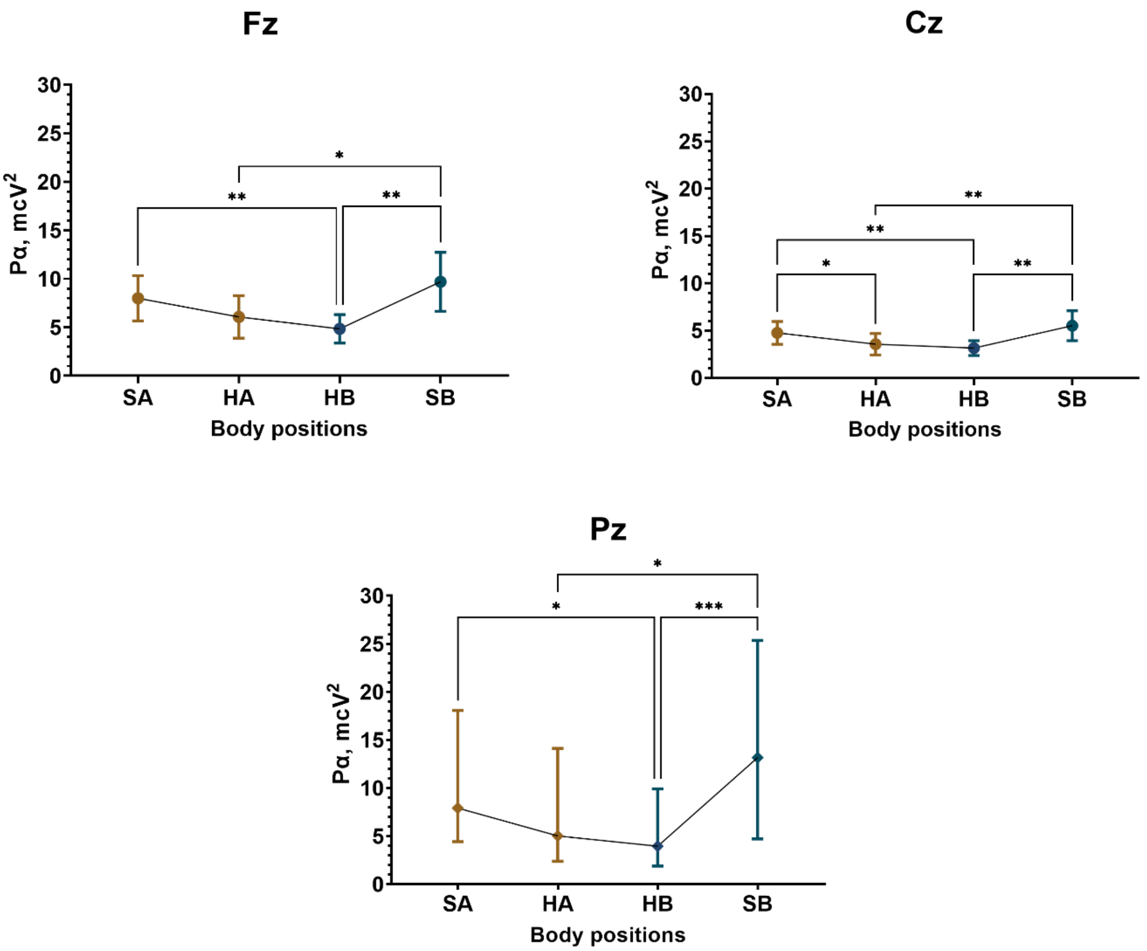

Supplemental Figure 40. Postural changes of alpha spectral power (Pα) calculated for Fz, Cz and Pz electrodes among male participants during Test 1 (n = 19). The graphs show confidence intervals with means represented by circle-shaped points, and medians depicted as rhomb-shaped points. Additionally, points and intervals are highlighted by different colors to distinguish between first sitting (SA) and first 2 minutes of supine (HA) position and second sitting (SB) and last 2 minutes of supine (HB) position. A one-way repeated measures ANOVA and a nonparametric Friedman test summaries for statistically significant results: Fz (F (1.864, 33.55) = 10.67, $p = 0.0003$), Cz (F (2.353, 42.35) = 11.67, $p < 0.0001$), Pz (*Friedman statistic* = 21.76, $p < 0.0001$). "*" – $p < 0.05$, "**" – $p < 0.01$, "***" – $p < 0.001$.
