# Supplementary material for: The role of cerebral blood flow volume in cortical inhibition during postural changes
Source: PeerJ. 2025 Oct 27;13:e20233. doi: 10.7717/peerj.20233 (PMC12574591; doi:10.7717/peerj.20233)
Supplement: Supplemental Information 50 — The graphs show confidence intervals with means represented by circle-shaped points, and medians depicted as rhomb-shaped points. Additionally, points and intervals are highlighted by different colors to distinguish between first sitting (oSA) and supine (oHA) positions and second sitting (oSB) and supine (oHB) positions. [file peerj-13-20233-s050.pdf]

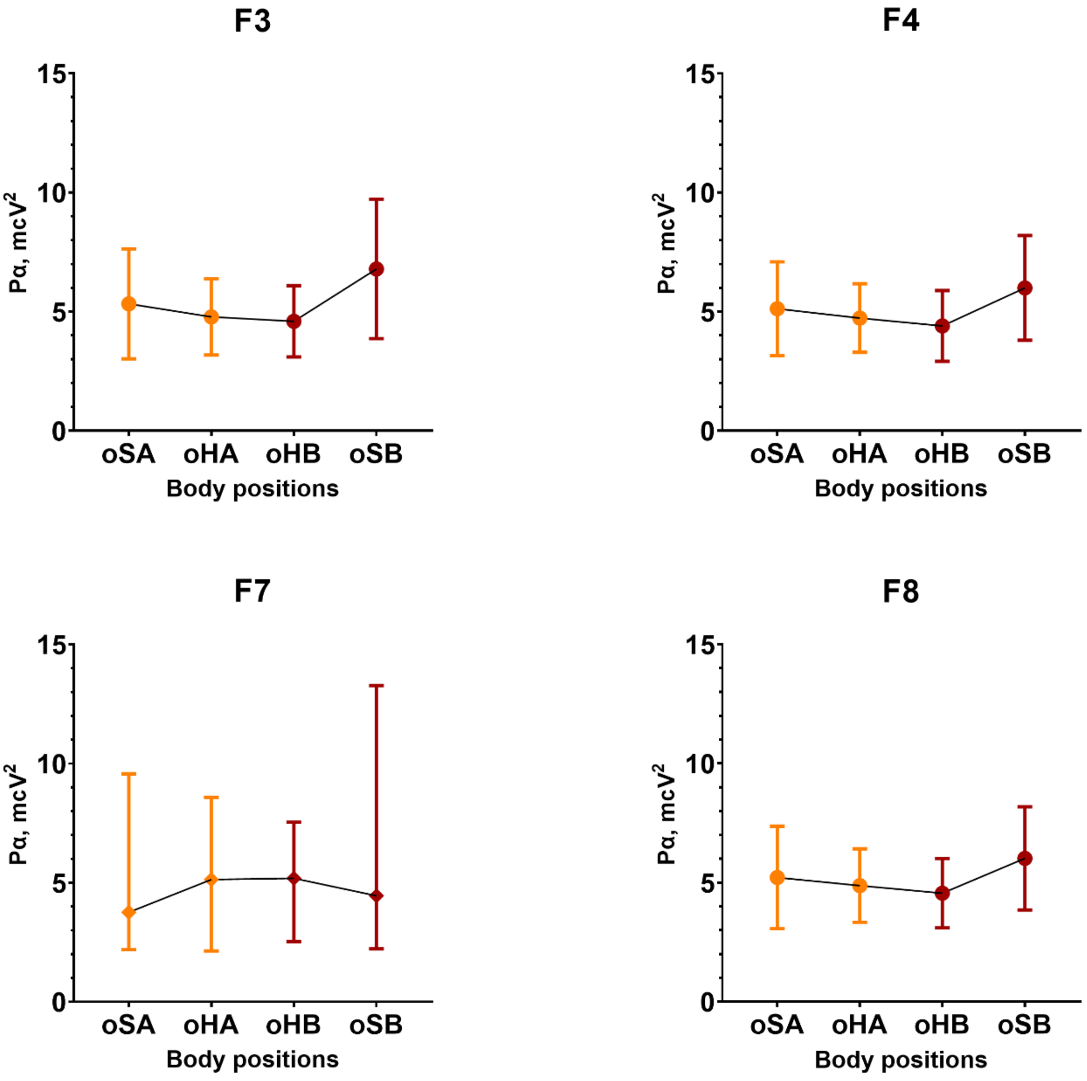


**Supplemental Figure 43. Postural changes of alpha spectral power (Pα) calculated for F3, F4, F7 and F8 electrodes among male participants during Test 2 (n = 16).** The graphs show confidence intervals with means represented by circle-shaped points, and medians depicted as rhomb-shaped points. Additionally, points and intervals are highlighted by different colors to distinguish between first sitting (oSA) and supine (oHA) positions and second sitting (oSB) and supine (oHB) positions.
