# Supplementary material for: The role of cerebral blood flow volume in cortical inhibition during postural changes
Source: PeerJ. 2025 Oct 27;13:e20233. doi: 10.7717/peerj.20233 (PMC12574591; doi:10.7717/peerj.20233)
Supplement: Supplemental Information 51 — The graphs show confidence intervals with means represented by circle-shaped points, and medians depicted as rhomb-shaped points. Additionally, points and intervals are highlighted by different colors to distinguish between first sitting (oSA) and supine (oHA) positions and second sitting (oSB) and supine (oHB) positions. A one-way repeated measures ANOVA and a nonparametric Friedman test summaries for statistically significant results: F3 (F (2.125, 34.00) = 3.569, p = 0.0367), F7 (Friedman statistic = 9.918, p = 0.0193), F8 (Friedman statistic = 9.494, p < 0.0234). “*” –p < 0.05. [file peerj-13-20233-s051.pdf]

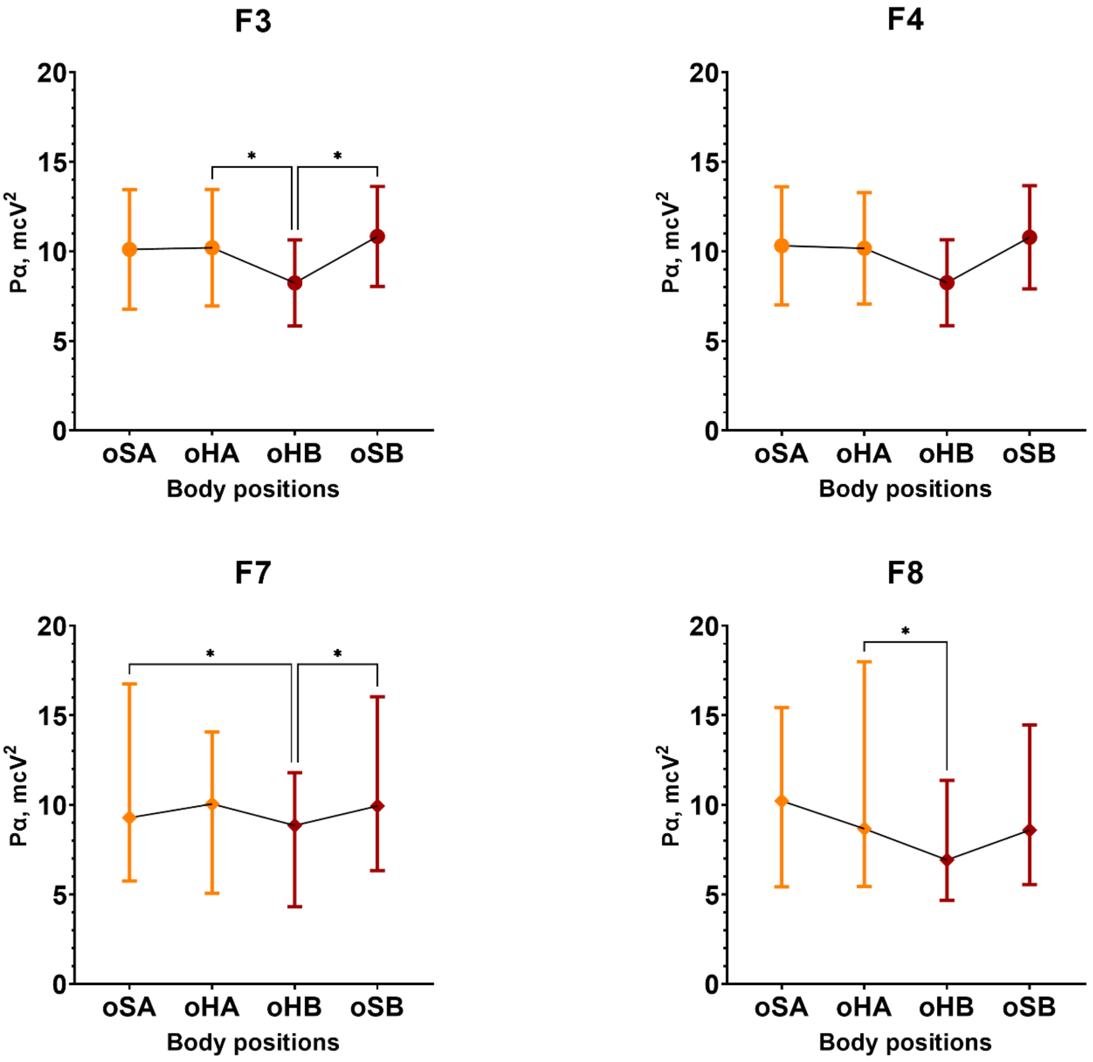

Supplemental Figure 44. Postural changes of alpha spectral power (Pα) calculated for F3, F4, F7 and F8 electrodes among female participants during Test 2 (n = 17). The graphs show confidence intervals with means represented by circle-shaped points, and medians depicted as rhomb-shaped points. Additionally, points and intervals are highlighted by different colors to distinguish between first sitting (oSA) and supine (oHA) positions and second sitting (oSB) and supine (oHB) positions. A one-way repeated measures ANOVA and a nonparametric Friedman test summaries for statistically significant results: F3 (F (2.125, 34.00) = 3.569, p = 0.0367), F7 (*Friedman statistic* = 9.918, p = 0.0193), F8 (*Friedman statistic* = 9.494, p < 0.0234). "*" – p < 0.05.
