# Supplementary material for: The role of cerebral blood flow volume in cortical inhibition during postural changes
Source: PeerJ. 2025 Oct 27;13:e20233. doi: 10.7717/peerj.20233 (PMC12574591; doi:10.7717/peerj.20233)
Supplement: Supplemental Information 52 — The graphs show confidence intervals with means represented by circle-shaped points, and medians depicted as rhomb-shaped points. Additionally, points and intervals are highlighted by different colors to distinguish between first sitting (oSA) and supine (oHA) positions and second sitting (oSB) and supine (oHB) positions. A one-way repeated measures ANOVA and a nonparametric Friedman test summaries for statistically significant results: F3 (F (2.302, 69.05) = 5.448, p = 0.0044), F7 (F (2.336, 72.4) = 4.329, p = 0.0126). “*” –p < 0.05, “**” –p < 0.01. [file peerj-13-20233-s052.pdf]

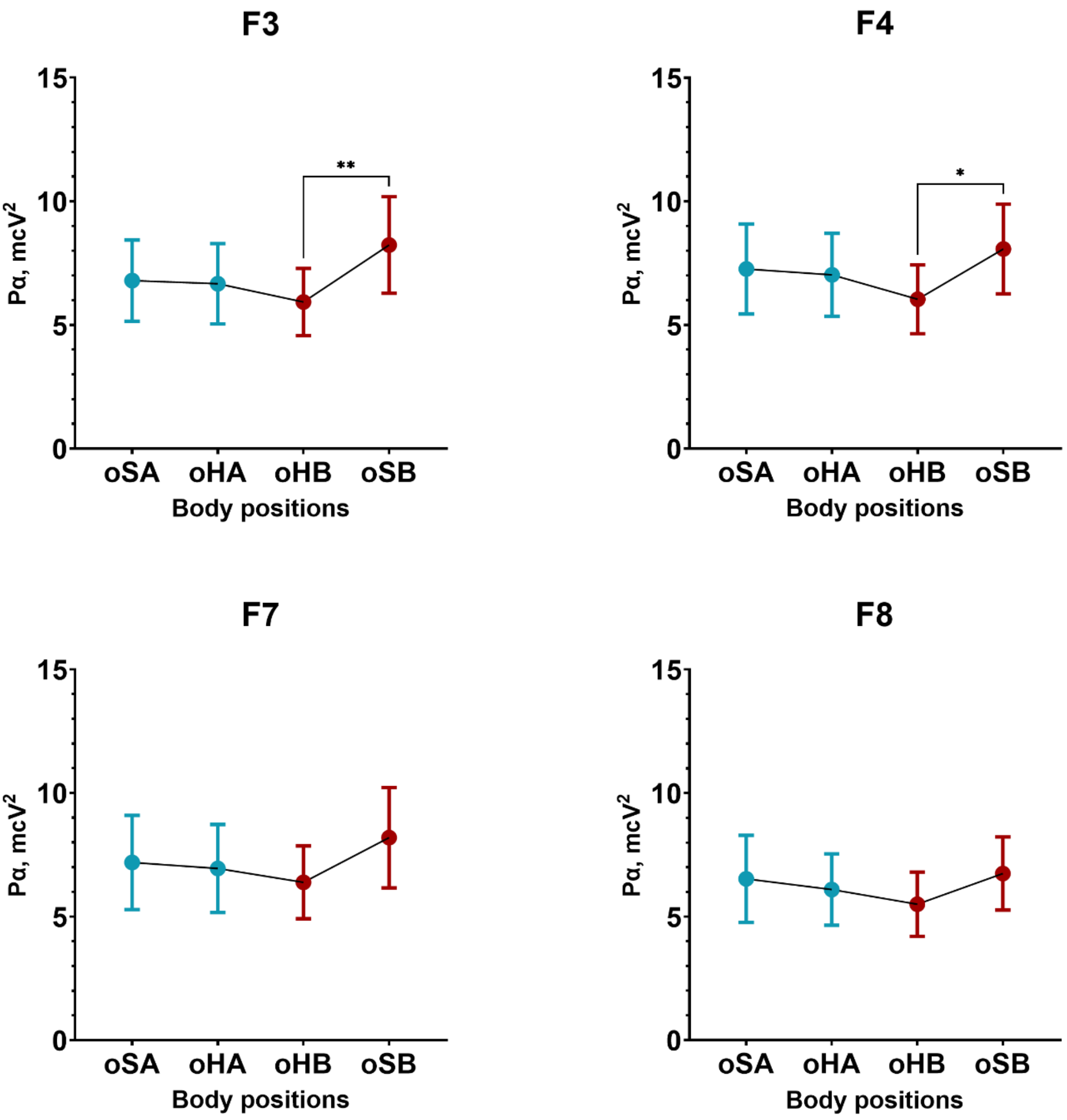

Supplemental Figure 45. Postural changes of alpha spectral power (Pα) calculated for F3, F4, F7 and F8 electrodes among all participants during Test 2 (n = 30). The graphs show confidence intervals with means represented by circle-shaped points, and medians depicted as rhomb-shaped points. Additionally, points and intervals are highlighted by different colors to distinguish between first sitting (oSA) and supine (oHA) positions and second sitting (oSB) and supine (oHB) positions. A one-way repeated measures ANOVA and a nonparametric Friedman test summaries for statistically significant results: F3 (F (2.302, 69.05) = 5.448, p = 0.0044), F7 (F (2.336, 72.4) = 4.329, p = 0.0126). "*" – $p < 0.05$, "**" – $p < 0.01$.
