# Supplementary material for: The role of cerebral blood flow volume in cortical inhibition during postural changes
Source: PeerJ. 2025 Oct 27;13:e20233. doi: 10.7717/peerj.20233 (PMC12574591; doi:10.7717/peerj.20233)
Supplement: Supplemental Information 53 — The graphs show confidence intervals with means represented by circle-shaped points, and medians depicted as rhomb-shaped points. Additionally, points and intervals are highlighted by different colors to distinguish between first sitting (oSA) and supine (oHA) positions and second sitting (oSB) and supine (oHB) positions. A one-way repeated measures ANOVA and a nonparametric Friedman test summaries for statistically significant results: C3 (F (2.075, 31.13) = 5.457, p = 0.0087), T4 (Friedman statistic = 8.925, p = 0.0303). “*” –p < 0.05. [file peerj-13-20233-s053.pdf]

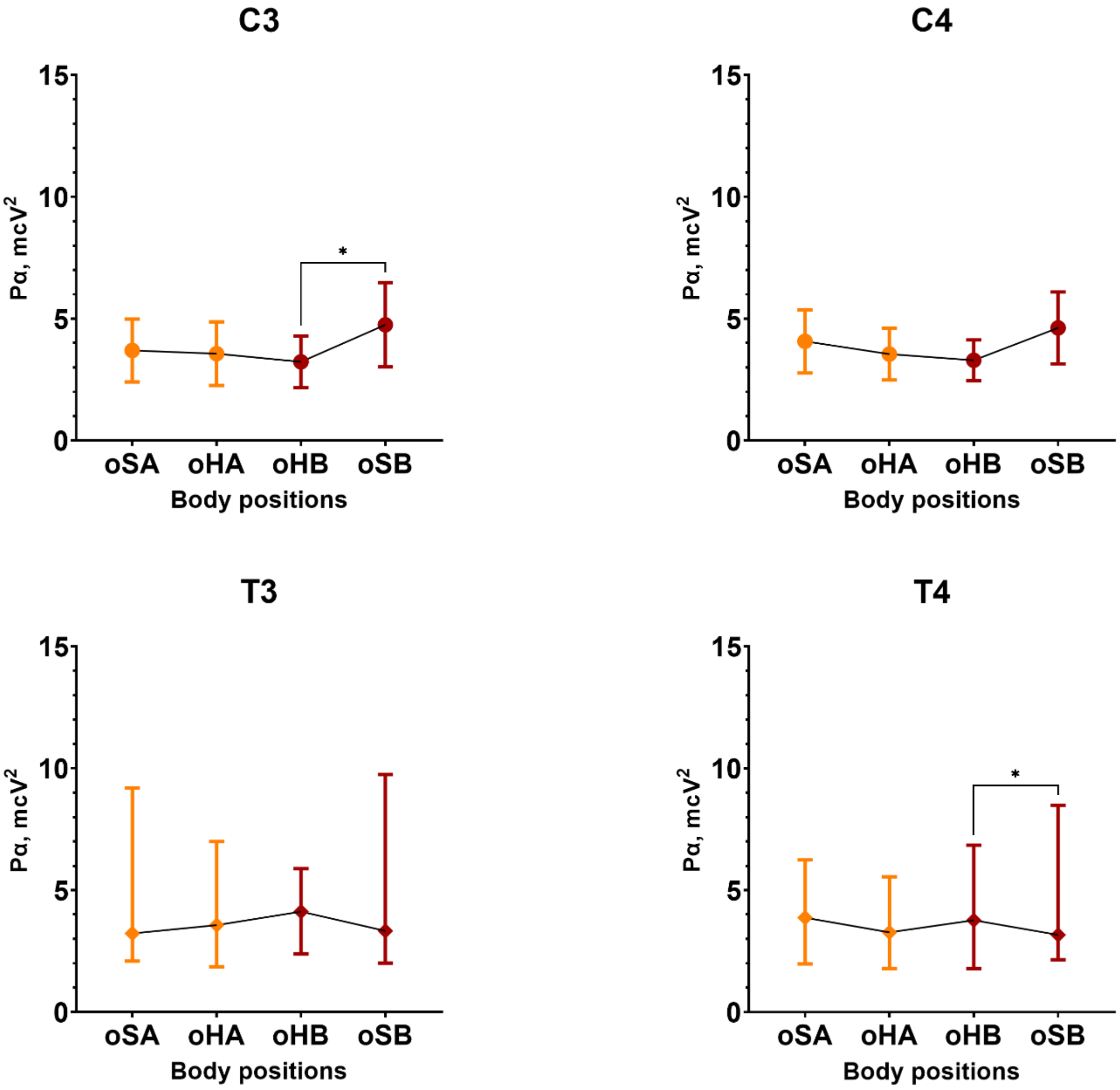

Supplemental Figure 46. Postural changes of alpha spectral power (Pα) calculated for C3, C4, T3 and T4 electrodes among male participants during Test 2 (n = 16). The graphs show confidence intervals with means represented by circle-shaped points, and medians depicted as rhomb-shaped points. Additionally, points and intervals are highlighted by different colors to distinguish between first sitting (oSA) and supine (oHA) positions and second sitting (oSB) and supine (oHB) positions. A one-way repeated measures ANOVA and a nonparametric Friedman test summaries for statistically significant results: C3 (F (2.075, 31.13) = 5.457, p = 0.0087), T4 (*Friedman statistic* = 8.925, p = 0.0303). "*" – $p < 0.05$.
