# Supplementary material for: The role of cerebral blood flow volume in cortical inhibition during postural changes
Source: PeerJ. 2025 Oct 27;13:e20233. doi: 10.7717/peerj.20233 (PMC12574591; doi:10.7717/peerj.20233)
Supplement: Supplemental Information 57 — The graphs show confidence intervals with means represented by circle-shaped points, and medians depicted as rhomb-shaped points. Additionally, points and intervals are highlighted by different colors to distinguish between first sitting (oSA) and supine (oHA) positions and second sitting (oSB) and supine (oHB) positions. A nonparametric Friedman test summary for statistically significant results: P4 (Friedman statistic = 15.56, p = 0.0014). “**” –p < 0.01. [file peerj-13-20233-s057.pdf]

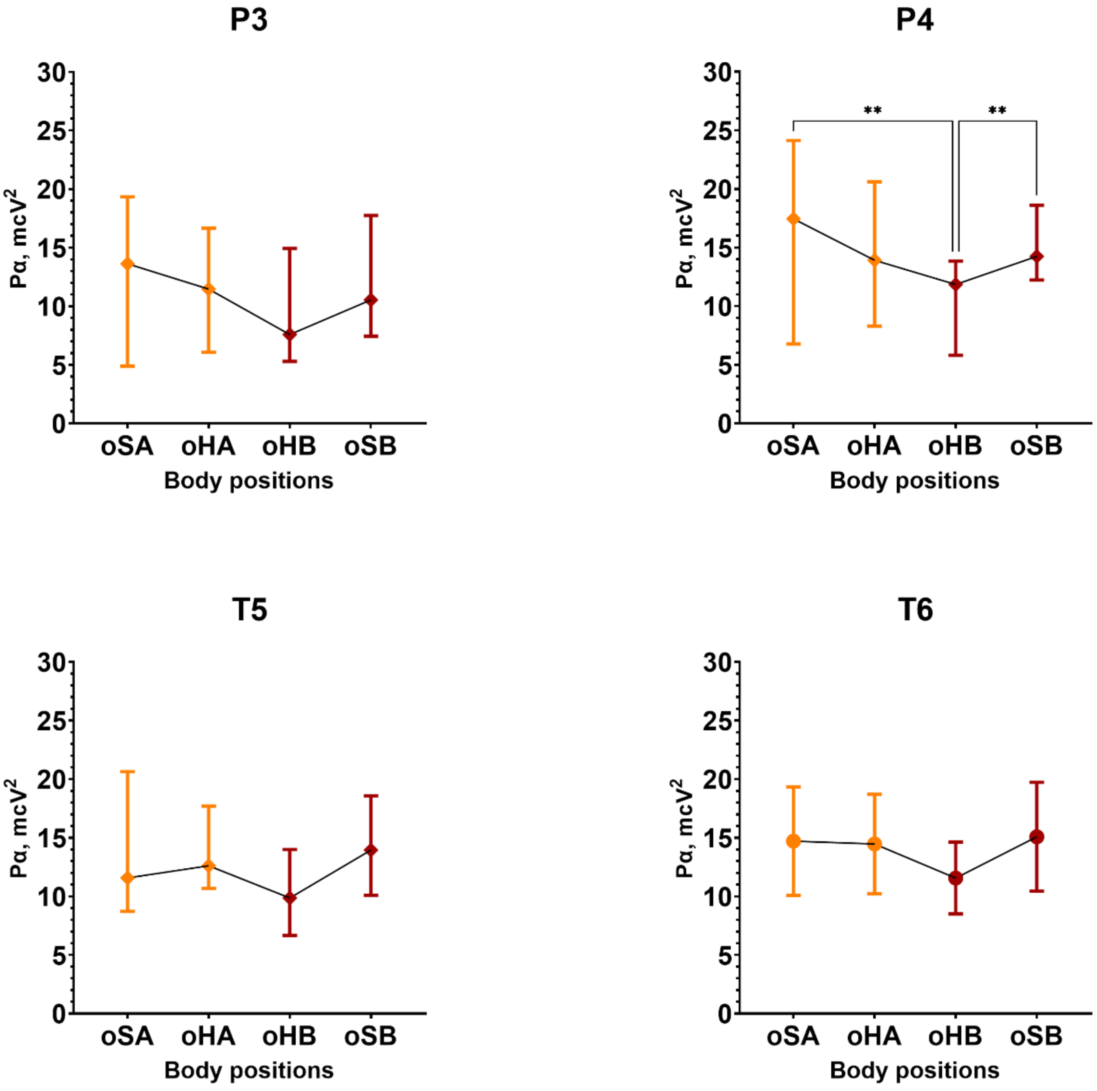

Supplemental Figure 50. Postural changes of alpha spectral power (Pα) calculated for P3, P4, T5 and T6 electrodes among female participants during Test 2 (n = 17). The graphs show confidence intervals with means represented by circle-shaped points, and medians depicted as rhomb-shaped points. Additionally, points and intervals are highlighted by different colors to distinguish between first sitting (oSA) and supine (oHA) positions and second sitting (oSB) and supine (oHB) positions. A nonparametric Friedman test summary for statistically significant results: P4 (*Friedman statistic* = 15.56, $p = 0.0014$). "**" – $p < 0.01$.
