# Supplementary material for: The role of cerebral blood flow volume in cortical inhibition during postural changes
Source: PeerJ. 2025 Oct 27;13:e20233. doi: 10.7717/peerj.20233 (PMC12574591; doi:10.7717/peerj.20233)
Supplement: Supplemental Information 58 — The graphs show confidence intervals with means represented by circle-shaped points, and medians depicted as rhomb-shaped points. Additionally, points and intervals are highlighted by different colors to distinguish between first sitting (oSA) and supine (oHA) positions and second sitting (oSB) and supine (oHB) positions. A nonparametric Friedman test summary for statistically significant results: P3 (Friedman statistic = 15.35, p = 0.0015), P3 (F (2.324, 65.07) = 4.614, p = 0.0099). “*” –p < 0.05, “**” –p < 0.01. [file peerj-13-20233-s058.pdf]

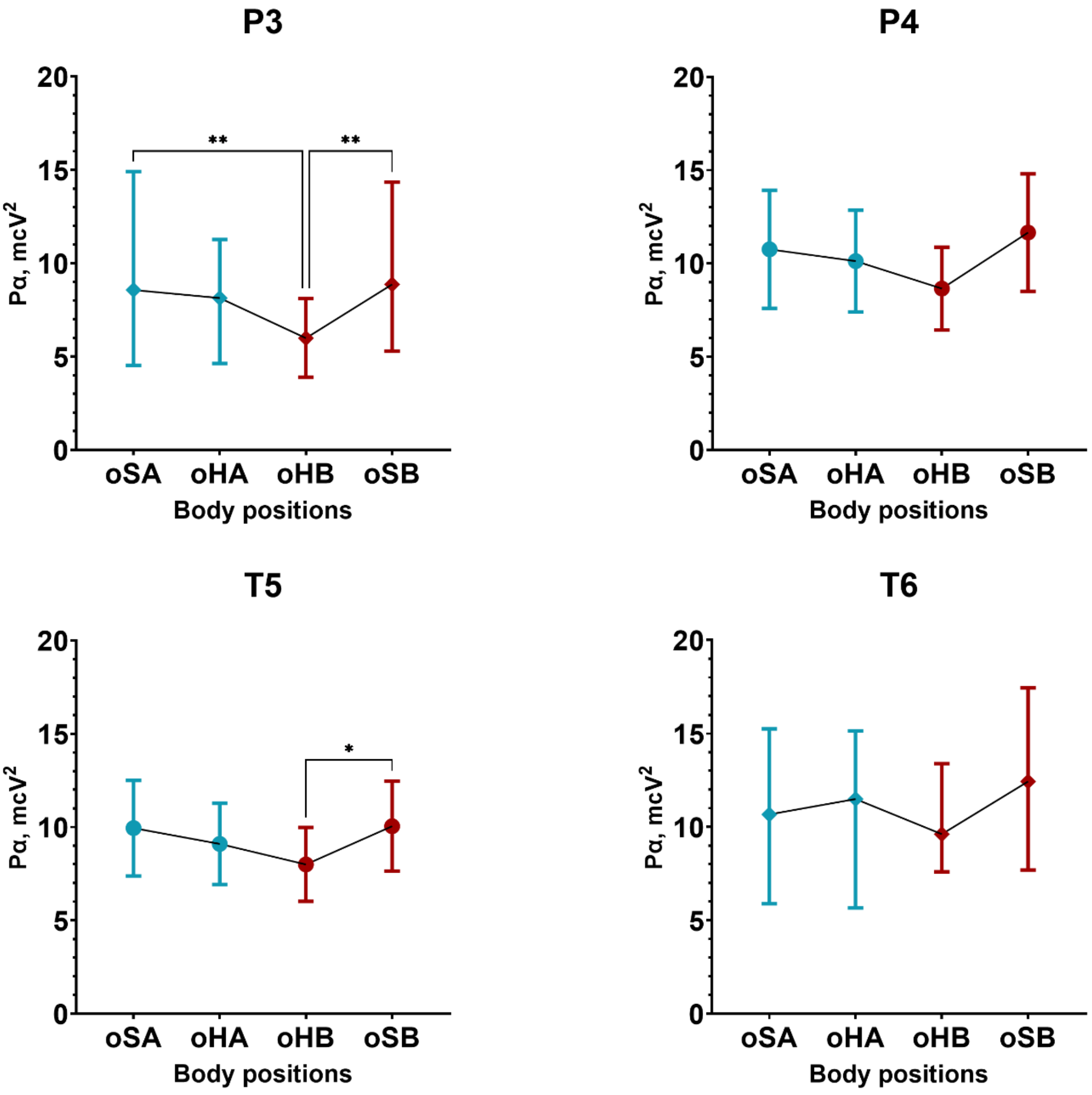

Supplemental Figure 51. Postural changes of alpha spectral power (Pα) calculated for P3, P4, T5 and T6 electrodes among all participants during Test 2 (n = 30). The graphs show confidence intervals with means represented by circle-shaped points, and medians depicted as rhomb-shaped points. Additionally, points and intervals are highlighted by different colors to distinguish between first sitting (oSA) and supine (oHA) positions and second sitting (oSB) and supine (oHB) positions. A nonparametric Friedman test summary for statistically significant results: P3 (*Friedman statistic* = 15.35, $p = 0.0015$), P3 (F (2.324, 65.07) = 4.614, $p = 0.0099$). "*" – p < 0.05, "**" – $p < 0.01$.
