# Supplementary material for: The role of cerebral blood flow volume in cortical inhibition during postural changes
Source: PeerJ. 2025 Oct 27;13:e20233. doi: 10.7717/peerj.20233 (PMC12574591; doi:10.7717/peerj.20233)
Supplement: Supplemental Information 60 — The graphs show confidence intervals with means represented by circle-shaped points, and medians depicted as rhomb-shaped points. Additionally, points and intervals are highlighted by different colors to distinguish between first sitting (oSA) and supine (oHA) positions and second sitting (oSB) and supine (oHB) positions. A one-way repeated measures ANOVA and a nonparametric Friedman test summaries for statistically significant results: Fz (F (2.085, 33.36) = 3.764, p = 0.032), Cz (Friedman statistic = 13.28, p = 0.0041). “*” –p < 0.05, “**” –p < 0.01. [file peerj-13-20233-s060.pdf]

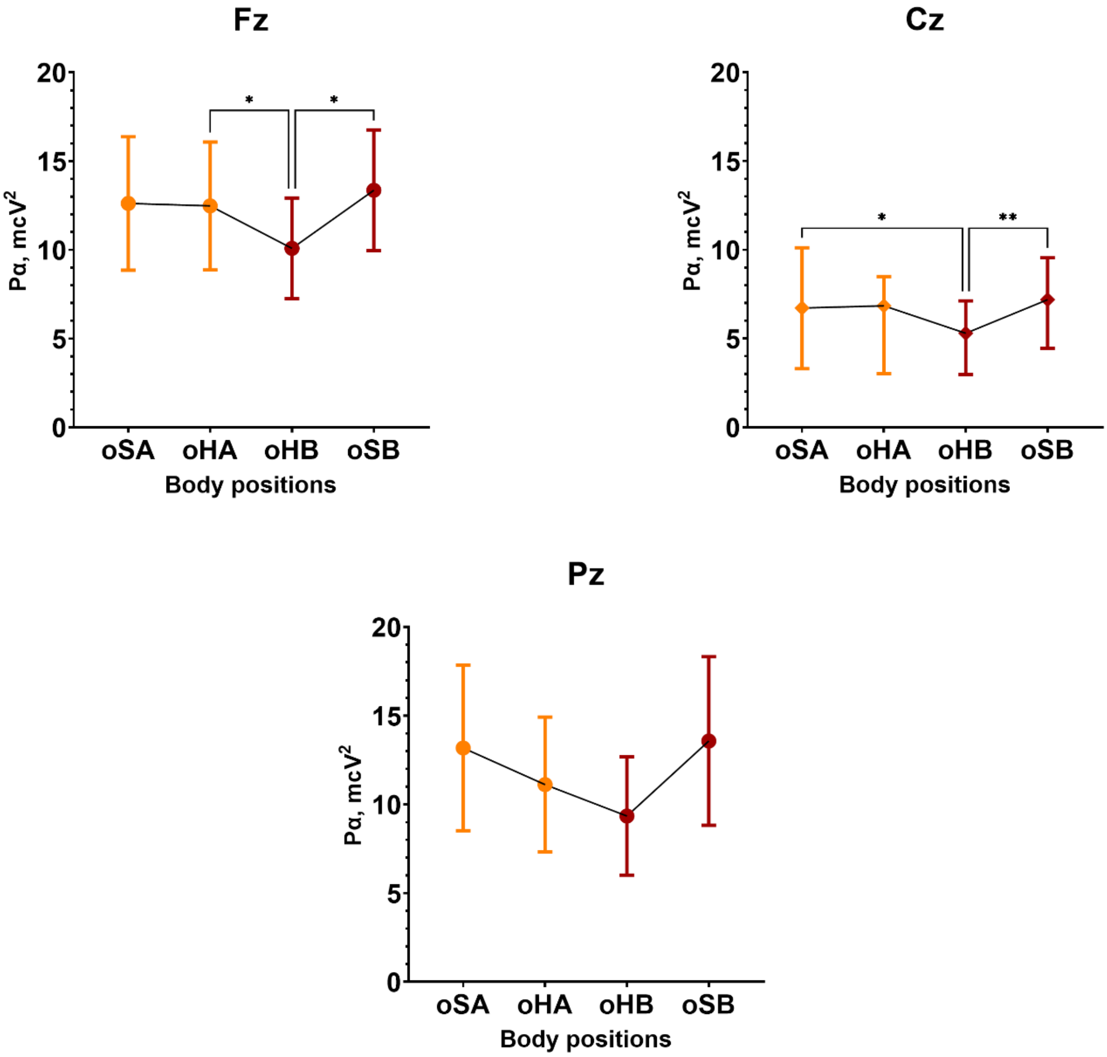

Supplemental Figure 53. Postural changes of alpha spectral power (Pα) calculated for Fz, Cz and Pz electrodes among female participants during Test 2 (n = 17). The graphs show confidence intervals with means represented by circle-shaped points, and medians depicted as rhomb-shaped points. Additionally, points and intervals are highlighted by different colors to distinguish between first sitting (oSA) and supine (oHA) positions and second sitting (oSB) and supine (oHB) positions. A one-way repeated measures ANOVA and a nonparametric Friedman test summaries for statistically significant results: Fz (F (2.085, 33.36) = 3.764, $p = 0.032$), Cz (*Friedman statistic* = 13.28, $p = 0.0041$). "*" – $p < 0.05$, "**" – $p < 0.01$.
