# Supplementary material for: The role of cerebral blood flow volume in cortical inhibition during postural changes
Source: PeerJ. 2025 Oct 27;13:e20233. doi: 10.7717/peerj.20233 (PMC12574591; doi:10.7717/peerj.20233)
Supplement: Supplemental Information 61 — The graphs show confidence intervals with means represented by circle-shaped points, and medians depicted as rhomb-shaped points. Additionally, points and intervals are highlighted by different colors to distinguish between first sitting (oSA) and supine (oHA) positions and second sitting (oSB) and supine (oHB) positions. A one-way repeated measures ANOVA and a nonparametric Friedman test summaries for statistically significant results: Fz (F (2.249, 71.96) = 5.319, p = 0.0052), Cz (Friedman statistic = 20.21, p = 0.0002), Pz (Friedman statistic = 14.92, p = 0.0019) “*” –p < 0.05, “**” –p < 0.01, “***” –p < 0.001. [file peerj-13-20233-s061.pdf]

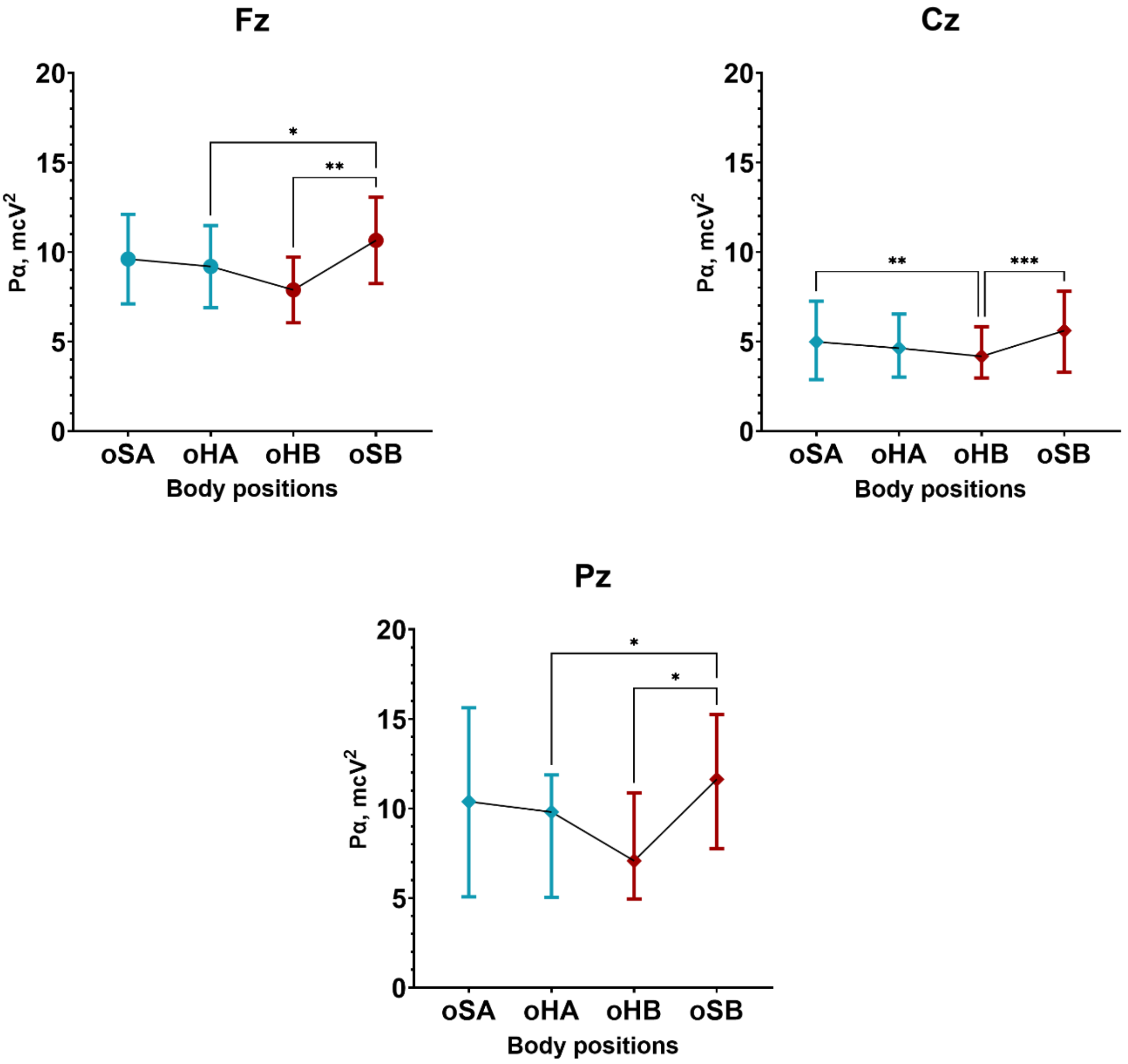

Supplemental Figure 54. Postural changes of alpha spectral power (Pα) calculated for Fz, Cz and Pz electrodes among all participants during Test 2 (n = 33). The graphs show confidence intervals with means represented by circle-shaped points, and medians depicted as rhomb-shaped points. Additionally, points and intervals are highlighted by different colors to distinguish between first sitting (oSA) and supine (oHA) positions and second sitting (oSB) and supine (oHB) positions. A one-way repeated measures ANOVA and a nonparametric Friedman test summaries for statistically significant results: Fz (F (2.249, 71.96) = 5.319, $p = 0.0052$), Cz (*Friedman statistic* = 20.21, $p = 0.0002$), Pz (*Friedman statistic* = 14.92, $p = 0.0019$) "*" – $p < 0.05$, "**" – $p < 0.01$, "***" – $p < 0.001$.
