# Supplementary material for: The role of cerebral blood flow volume in cortical inhibition during postural changes
Source: PeerJ. 2025 Oct 27;13:e20233. doi: 10.7717/peerj.20233 (PMC12574591; doi:10.7717/peerj.20233)
Supplement: Supplemental Information 62 — Black boxplots include values of male participants (m), and red boxplots contain values of female participants (f). Pairs of boxplots were analyzed separately, i.e., HA (m) was compared only to HA (f), and HB (m) was compared only to HB (f). Outliers are shown by black and blue points. A one-way ANOVA and a nonparametric Kruskal–Wallis test summaries for statistically significant results: F3 (F (3, 66) = 6.412, p = 0.0007), F4 (F (3, 66) = 6.543, p = 0.0006), C3 (F (3, 66) = 6.562, p = 0.0006), C4 (Kruskal–Wallis statistic = 12.87, p = 0.0049), P3 (Kruskal–Wallis statistic = 10.07, p = 0.018), P4 (Kruskal–Wallis statistic = 12.45, p = 0.006) “*” –p < 0.05, “**” –p < 0.01. [file peerj-13-20233-s062.pdf]

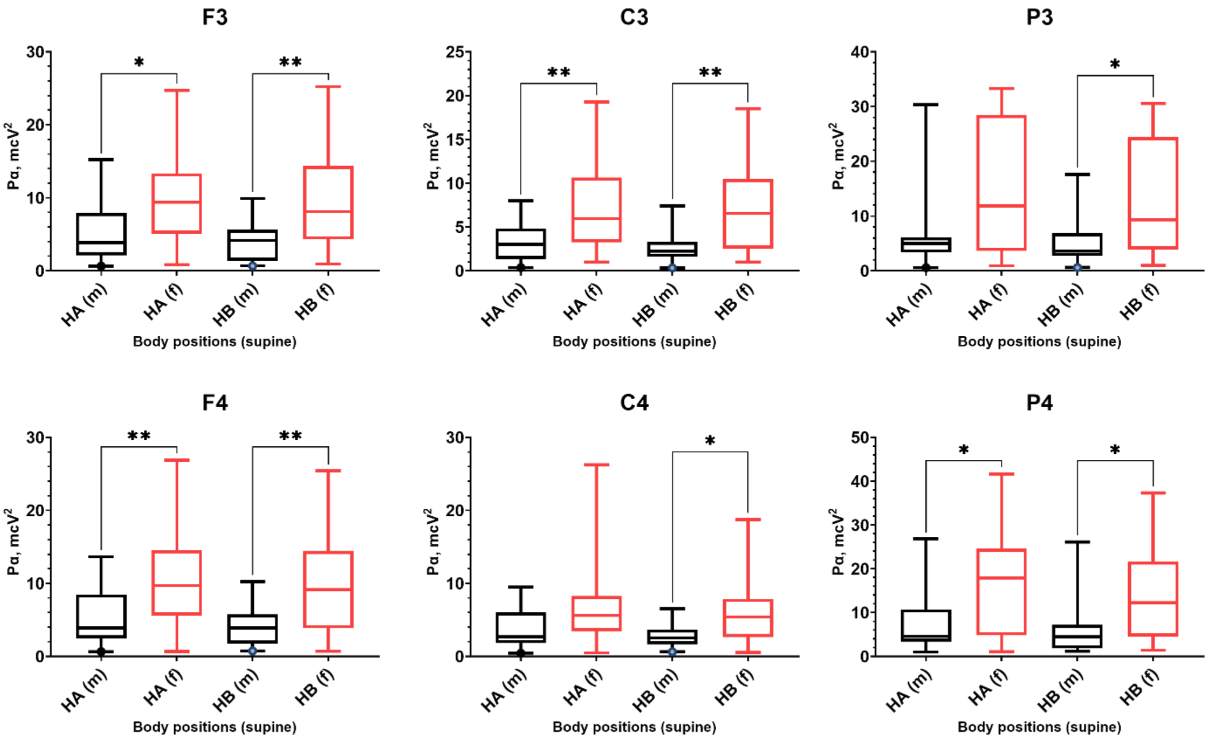

Supplemental Figure 55. Sex differences in alpha spectral power (Pα) for F3, F4, C3, C4, P3 and P4 electrodes during first (HA) and last (HB) 2 minutes of supine position in Test 1 (*n* = 35). Black boxplots include values of male participants (m), and red boxplots contain values of female participants (f). Pairs of boxplots were analyzed separately, i.e., HA (m) was compared only to HA (f), and HB (m) was compared only to HB (f). Outliers are shown by black and blue points. A one-way ANOVA and a nonparametric Kruskal-Wallis test summaries for statistically significant results: F3 (F (3, 66) = 6.412, p = 0.0007), F4 (F (3, 66) = 6.543, p = 0.0006), C3 (F (3, 66) = 6.562, p = 0.0006), C4 (*Kruskal-Wallis statistic* = 12.87, p = 0.0049), P3 (*Kruskal-Wallis statistic* = 10.07, p = 0.018), P4 (*Kruskal-Wallis statistic* = 12.45, p = 0.006) "*" – p < 0.05, "**" – p < 0.01.
