# Supplementary material for: The role of cerebral blood flow volume in cortical inhibition during postural changes
Source: PeerJ. 2025 Oct 27;13:e20233. doi: 10.7717/peerj.20233 (PMC12574591; doi:10.7717/peerj.20233)
Supplement: Supplemental Information 66 — Black boxplots include values of male participants (m), and red boxplots contain values of female participants (f). Pairs of boxplots were analyzed separately, i.e., oHA (m) was compared only to oHA (f), and oHB (m) was compared only to oHB (f). A one-way ANOVA and a nonparametric Kruskal–Wallis test summaries for statistically significant results: F7 (F (3, 62) = 5.622, p = 0.0018), F8 (Kruskal–Wallis statistic = 13.76, p = 0.0033), T3 (F (3, 60) = 4.495, p = 0.0065), T4 (F (3, 60) = 4.884, p = 0.0042), T5 (Kruskal–Wallis statistic = 13.27, p = 0.0041). “*” –p < 0.05, “**” –p < 0.01. [file peerj-13-20233-s066.pdf]

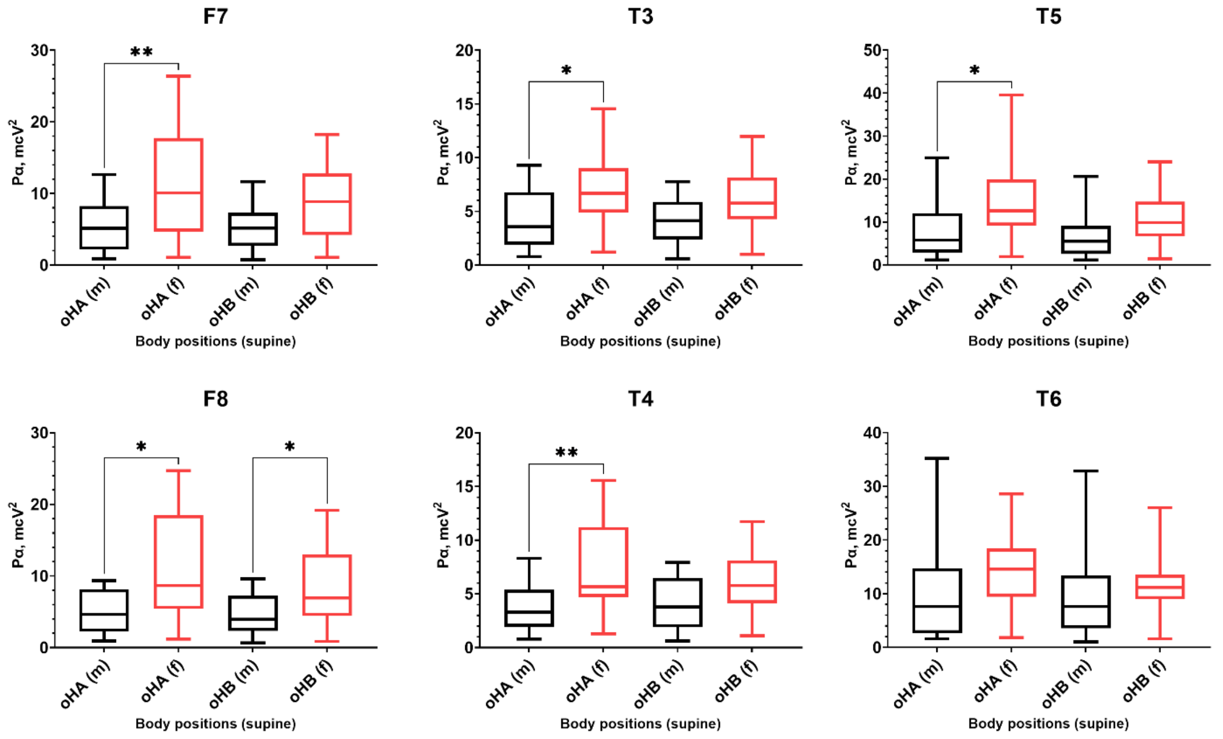


**Supplemental Figure 59. Sex differences in alpha spectral power (Pα) for F7, F8, T3, T4, T5 and T6 electrodes during supine positions (oHA and oHB) in Test 2 (*n* = 33).** Black boxplots include values of male participants (m), and red boxplots contain values of female participants (f). Pairs of boxplots were analyzed separately, i.e., oHA (m) was compared only to oHA (f), and oHB (m) was compared only to oHB (f). A one-way ANOVA and a nonparametric Kruskal-Wallis test summaries for statistically significant results: F7 ($F$ (3, 62) = 5.622, $p$ = 0.0018), F8 (*Kruskal-Wallis statistic* = 13.76, $p$ = 0.0033), T3 ($F$ (3, 60) = 4.495, $p$ = 0.0065), T4 ($F$ (3, 60) = 4.884, $p$ = 0.0042), T5 (*Kruskal-Wallis statistic* = 13.27, $p$ = 0.0041). "*" – $p < 0.05$, "**" – $p < 0.01$.
