# Supplementary material for: The role of cerebral blood flow volume in cortical inhibition during postural changes
Source: PeerJ. 2025 Oct 27;13:e20233. doi: 10.7717/peerj.20233 (PMC12574591; doi:10.7717/peerj.20233)
Supplement: Supplemental Information 70 — Each point of certain color represents estimated relationship between RWA and Pα in the corresponding pair of REG lead and EEG electrode, for example, left FM and F3 (LFM-F3), right OM and C4 (RFM-C4), etc. The lines indicate the general trend of the relationship. [file peerj-13-20233-s070.pdf]

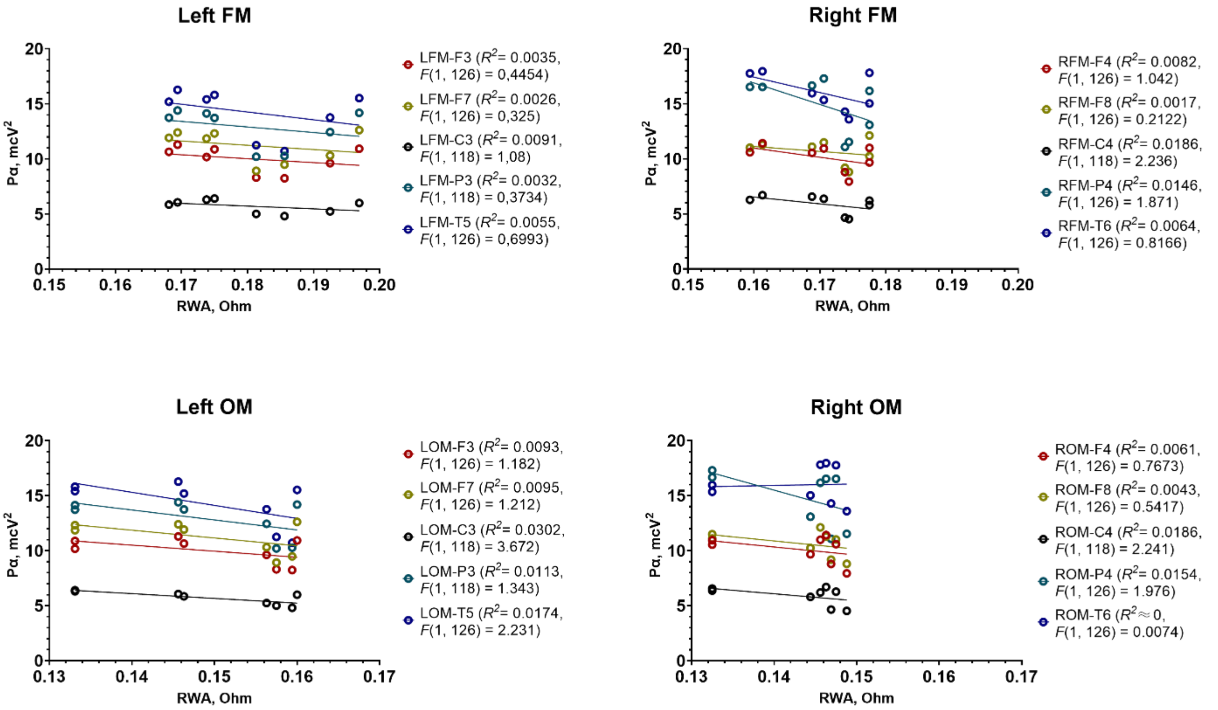

Suplemental Figure 63. Simple linear regression graphs showing estimated relationship between RWA and Pα in all 4 REG leads and several corresponding EEG electrodes among female participants in Test 2 (*n* = 8). Each point of certain color represents estimated relationship between RWA and Pα in the corresponding pair of REG lead and EEG electrode, for example, left FM and F3 (LFM-F3), right OM and C4 (RFM-C4), etc. The lines indicate the general trend of the relationship.
